# Development of a novel selection/counter-selection system for chromosomal gene integrations and deletions in lactic acid bacteria

**DOI:** 10.1186/s12867-019-0127-x

**Published:** 2019-03-29

**Authors:** Winschau F. Van Zyl, Leon M. T. Dicks, Shelly M. Deane

**Affiliations:** Department of Microbiology, Stellenbosch University, Stellenbosch, 7600 South Africa

**Keywords:** Homologous recombination, MazF toxin, Counter-selection, Gene insertion/deletion, Antibiotic marker recycling

## Abstract

**Background:**

The underlying mechanisms by which probiotic lactic acid bacteria (LAB) enhance the health of the consumer have not been fully elucidated. Verification of probiotic modes of action can be achieved by using single- or multiple-gene knockout analyses of bacterial mutants in in vitro or in vivo models. We developed a novel system based on an inducible toxin counter-selection system, allowing for rapid and efficient isolation of LAB integration or deletion mutants. The *Lactococcus lactis* nisin A inducible promoter was used for expression of the *Escherichia coli mazF* toxin gene as counter-selectable marker.

**Results:**

The flippase (FLP)/flippase recognition target (FRT) recombination system and an antisense RNA transcript were used to create markerless chromosomal gene integrations/deletions in LAB. Expression of NisR and NisK signalling proteins generated stable DNA integrations and deletions. Large sequences could be inserted or deleted in a series of steps, as demonstrated by insertion of the firefly bioluminescence gene and erythromycin resistance marker into the bacteriocin operons or adhesion genes of *Lactobacillus plantarum* 423 and *Enterococcus mundtii* ST4SA.

**Conclusions:**

The system was useful in the construction of *L. plantarum* 423 and *E. mundtii* ST4SA bacteriocin and adhesion gene mutants. This provides the unique opportunity to study the role of specific probiotic LAB genes in complex environments using reverse genetics analysis. Although this work focuses on two probiotic LAB strains, *L*. *plantarum* 423 and *E. mundtii* ST4SA, the system developed could be adapted to most, if not all, LAB species.

**Electronic supplementary material:**

The online version of this article (10.1186/s12867-019-0127-x) contains supplementary material, which is available to authorized users.

## Background

In recent years a profitable probiotic market has emerged, with an increasing number of probiotic-containing supplements conferring specific health benefits to the consumer [1]. Strains of lactic acid bacteria (LAB) and bifidobacteria are the most frequently used as probiotics, and form part of many functional food and dietary supplements [2–4]. Probiotic LAB have a long history of safe use in food and therapeutic products and there is an increased recognition of their beneficial effects on human health [5]. However, the underlying mechanisms responsible for these effects on the health of the consumer have yet to be fully elucidated and are likely to be multifactorial. If the growing consumer interest in probiotics is to continue, it is crucial to identify the precise mechanisms of action by which probiotics influence human health. One way to provide verification of probiotic modes of action is the use of single or multiple gene knockout analyses of bacterial strains in in vitro or in vivo models [6].

The functional analysis of proteins expressed by genes that confer specific phenotypic properties has underpinned biotechnology for decades. The potential scope of such an approach has grown exponentially with the availability of whole-genome sequencing, commercial de novo DNA synthesis and synthetic biology [7]. While many species of LAB are now transformable by electroporation and have thus become amenable to genetic manipulation using plasmid vectors, methods for the isolation of stable and irreversible genetic mutants are still underdeveloped [8–11]. The construction of tailor-made LAB strains for functional genetic analyses is dependent on efficient genetic methods and is often reliant on chromosomal integrations or deletions of specific target genes. This calls for the development of techniques that will allow for the easy and efficient selection of chromosomally- or plasmid-located gene excisions/integrations.

For many years, researchers have used replicative plasmids to express foreign DNA in microorganisms, but these are inherently unstable when expressed in vitro or in vivo where antibiotic selection is not possible, thus limiting their applied utility [12]. To circumvent antibiotic selection-related issues, exogenous DNA must be irreversibly incorporated into a DNA molecule inside the cell. In this way, integration of recombinant DNA is achieved by positioning a selectable marker gene alongside a DNA sequence that is homologous to the target gene of interest within an allele exchange cassette. This has been achieved successfully in yeast and naturally competent *Bacillus subtilis* that are easily transformable with linear DNA [13]. However, for most bacteria, including LAB, genetic engineering using linear DNA is a challenging task [14, 15]. Previous studies have demonstrated that genetic recombination in some LAB species using single stranded linear DNA (ssDNA) is possible and that high recombineering efficiencies can be achieved when combined with clustered, regularly interspaced, palindromic repeats (CRISPRs) and a CRISPR-associated (Cas) nuclease [14–16]. However, establishing ssDNA recombination in new species is not trivial and requires extensive optimization procedures to eliminate low recombination frequencies [14]. Consequently, integration plasmids bearing DNA homologous to sites of chromosomal integration may be used to generate desired gene deletions or insertions in the absence of antibiotic marker selection. Desired recombinant cells are specifically selected and isolated using the selectable marker, typically an antibiotic resistance gene. Several sequences can be inserted at multiple loci by simply alternating between selectable markers, usually antibiotic resistance genes, as described in the ‘domino’ method of Itaya et al. [17]. This method can be effective, but is not without limitations. A major drawback is the availability of suitable antibiotic resistance markers for use as selection/counter-selection markers in the strain of interest. According to this strategy, different antibiotic resistance genes have to be used to introduce multiple chromosomal modifications. Moreover, multi-antibiotic selection pressure could potentially modify the physiology of the recombinant strain or antibiotic genes could potentially be passed to other bacteria.

One method commonly used for the construction of stable integration mutants in LAB is the use of plasmid vectors containing homologous sequences to the chromosomally located conjugative transposon Tn919, which is utilized as the locus for insertion into the host genome [18, 19]. While this method has been successfully applied in *Enterococcus faecalis* and *Lactococcus lactis*, it cannot be used for specific targeted gene deletions. Another method often employed for targeted gene inactivation and DNA chromosomal integrations is based on the use of suicide plasmids [18–25]. In most of these studies, the isolation of successful integrations was straightforward, but strains that have had the plasmid backbone removed may be difficult to isolate. Moreover, single homologous recombination events may be unstable and reversible, resulting in single-crossover mutants with the potential to revert back to the wild-type (WT). This can be overcome by the use of a counter-selection marker located on the plasmid backbone, but the identification of a suitable counter-selection marker under specific conditions to use as a genetic tool can be a challenging task [26, 27]. The *upp* gene, which codes for uracil phosphoribosyltransferase has been used as a counter-selectable marker in *Lc. lactis* [28, 29]. The *upp* gene is responsible for conferring toxicity to cells in the presence of 5-fluorouracil (5-FU), whereas the loss thereof leads to resistance to 5-FU. The main limitation of using the *upp* gene as counter-selectable marker is that it is present in the nucleotide metabolic pathway of almost every organism [30]. Another disadvantage is that 5-FU may be toxic, even in *upp* mutants, thus further hampering its use as a heterologous counter-selectable marker. Nonetheless, counter-selectable markers have proven invaluable in the construction of clean and unmarked gene deletions or insertions in various unicellular microorganisms [26, 30–36]. For a review on the use of counter-selectable markers as genetic tools, refer [26].

This study reports on the development of a strategy generally applicable to all LAB species for the quick and efficient isolation of double-crossover homologously recombined mutants at any genomic loci. We describe the use of a toxin gene as counter-selectable marker placed under the control of the *Lc. lactis* nisin inducible promoter (PnisA) that forms part of the well-characterized nisin-controlled expression (NICE) system (for a complete review on the NICE expression system, see reference [37]). The PnisA promoter is auto-inducible by nisin in *Lc. lactis*, but can be induced heterologously in other LAB strains using sub-inhibitory concentrations of nisin [38, 39]. For heterologous exploitation of the NICE system, the *nisK* and *nisR* nisin regulatory genes required for signal transduction have to be expressed in conjunction with the use of the PnisA promoter [40]. The *E. coli mazF* gene was chosen as a toxin gene for plasmid-borne counter-selection. The *mazF* gene is an mRNA interferase (sequence-specific endoribonuclease) that forms part of the MazE-MazF toxin-antitoxin (TA) system of *E. coli*, encoded by the *mazEF* operon [41]. Ectopic expression of the MazF toxin promotes cell death by specifically targeting mRNAs at ACA sequences [42]. In the counter-selection system described here, the expression of the MazF toxin under the control of PnisA is directly coupled to the formation of desired double-crossover mutants using an associated selectable phenotype. Using this method, DNA fragments can be irreversibly inserted or deleted at any genomic locus in *L. plantarum* 423 and *E. mundtii* ST4SA in a step-by-step manner. The application of the FLP-FRT recombination system to generate markerless homologously recombined mutants is also demonstrated. This method is applicable to many, if not all, transformable LAB species.

## Results

### Construction and optimization of the nisin-inducible counter-selection marker system

**Fig. 1:**
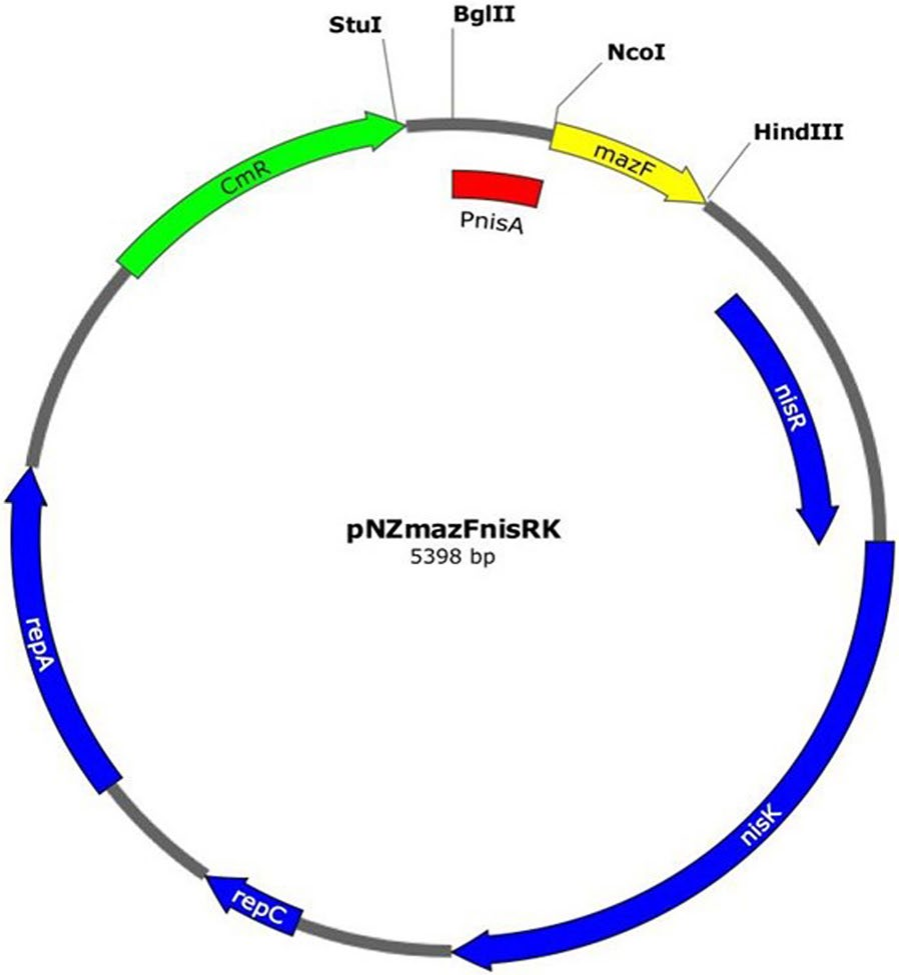
Schematic representing the pNZmazFnisRK inducible mazF toxin vector used for the construction of double-crossover homologously recombined DNA integration or deletion mutants at any genomic loci. Relevant features are indicated, including restriction sites used for cloning; the *E. coli*/LAB *repA* and *repC* replication genes; the chloramphenicol acetyltransferase (*cat*) gene conferring resistance to chloramphenicol; the *nisR* and *nisK* nisin regulatory genes; and the nisin-inducible PnisA promoter from *Lc. lactis* pNZ9000. Integration cassettes are inserted via blunting into the *Bgl*II, *Hin*dIII or *Stu*I restriction sites

**Fig. 2:**
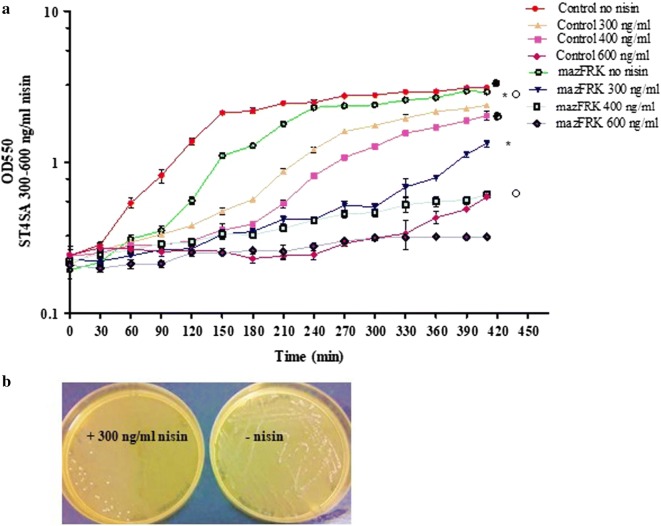
Optimization of nisin-controlled *mazF* gene expression in *E. mundtii* ST4SA. **a** Growth comparison of *E. mundtii* ST4SA transformed with the empty pNZ8048 vector (control) and *E. mundtii* ST4SA transformed with the PnisA-controlled *mazF* gene pNZmazFnisRK plasmid in sub-inhibitory concentrations of nisin (0–600 ng/ml). Significant differences were assessed using the Kruskal–Wallis nonparametric test, and are indicated with a filled circle (*P *< 0.05) for comparison between Control no nisin and control 400 ng/ml, an asterisk (*P *< 0.05) for comparison between mazFRK no nisin and mazFRK 300 ng/ml and an empty circle (*P *< 0.05) for comparison between mazFRK no nisin and mazFRK 400 ng/ml. **b** MRS agar plates representative of the effect of MazF protein expression in *E. mundtii* ST4SA harboring the pNZmazFnisRK plasmid in the absence of nisin (−nisin) and in the presence of nisin (+300 ng/ml nisin)

The heterologous use of PnisA originating from the *Lc. lactis* nisin A lantibiotic producer strain was optimized for the probiotics *L. plantarum* 423 and *E. mundtii* ST4SA. The plasmid pNZmazFnisRK (Fig. 1) containing the *mazF* toxin gene under control of the *Lc. lactis* nisin-inducible promoter, together with the regulatory genes, *nisR* and *nisK*, was introduced into the probiotic LAB strains, giving rise to *E. mundtii* ST4SA pNZmazFnisRK and *L. plantarum* 423 pNZmazFnisRK. Their MazF response to induction with varying concentrations of nisin was then evaluated (Fig. 2 and Additional file 1: Fig. S1). Previous studies [37, 38] reported that low sub-inhibitory concentrations of nisin, ranging from 0.1 to 5.0 ng/ml, were sufficient for PnisA-controlled gene expression in *Lc. lactis* pNZ9000. We found that the PnisA promoter responded to much higher sub-inhibitory concentrations of nisin, ranging from 300 to 400 ng/ml in *E. mundtii* ST4SA pNZmazFnisRK (Fig. 2a) and 300 to 600 ng/ml nisin in *L. plantarum* 423 pNZmazFnisRK after 7 h of induction (see Additional file 1: Fig. S1). Control strains transformed with pNZ8048 and recombinant strains transformed with pNZmazFnisRK without nisin induction showed no significant differences in growth after 7 h. This suggested that no significant promoter leakiness was occurring that could potentially result in premature cell growth arrest without nisin induction of PnisA. No significant difference in growth rate was recorded for control strain *E. mundtii* ST4SA pNZ8048 with (300 ng/ml) or without nisin induction, whereas a significant growth difference was recorded for recombinant *E. mundtii* ST4SA pNZmazFnisRK when induced with nisin at the same concentration (Fig. 2a). No significant growth inhibition was observed for control strain *L. plantarum* 423 pNZ8048 when induced with nisin concentrations ranging from 100 to 600 ng/ml compared to un-induced. A significant inhibition of growth was recorded when strain *L. plantarum* 423 pNZmazFnisRK was induced with nisin at 600 ng/ml as compared to when no nisin was present (see Additional file 1: Fig. S1). For all further nisin induction experiments, a nisin induction concentration of 300 ng/ml was used for PnisA-carrying *E. mundtii* ST4SA and 600 ng/ml for *L. plantarum* 423. Taken together, these results confirmed that the MazF protein toxicity would enable its application in LAB as an efficient counter-selection marker for the isolation of double-crossover mutants.

*Lactococcus lactis* pNZ9000 transformed with plasmid pNZmazF, and recombinant strains *L. plantarum* 423 pNZmazFnisRK and *E. mundtii* ST4SA pNZmazFnisRK were plated onto M17 or MRS agar plates supplemented with chloramphenicol (Cm) in the absence and presence of nisin. After 24 h of incubation at 30 °C, plates containing nisin showed very little or no growth, while those without nisin showed an abundance of growth (Fig. 2b, Additional file 1: Fig S2). To further demonstrate the application of the nisin-induction system in LAB, *Lc. lactis* pNZ9000 was transformed with plasmid pNZCherry, while *L. plantarum* 423 and *E. mundtii* ST4SA were transformed with plasmid pNZCherrynisRK. In both vectors, the *mCherry* red fluorescence gene was placed downstream of the PnisA promoter (Additional file 1: Table S1). The PnisA nisin-induced expression of mCherry fluorescence protein was easily detected on agar plates containing nisin by the appearance of pink colonies, while those lacking nisin remained white or cream (see Additional file 1: Fig. S2).

### Integration of large DNA fragments and deletion of the *L. plantarum* 423 and *E. mundtii* ST4SA bacteriocin genes

**Fig. 3:**
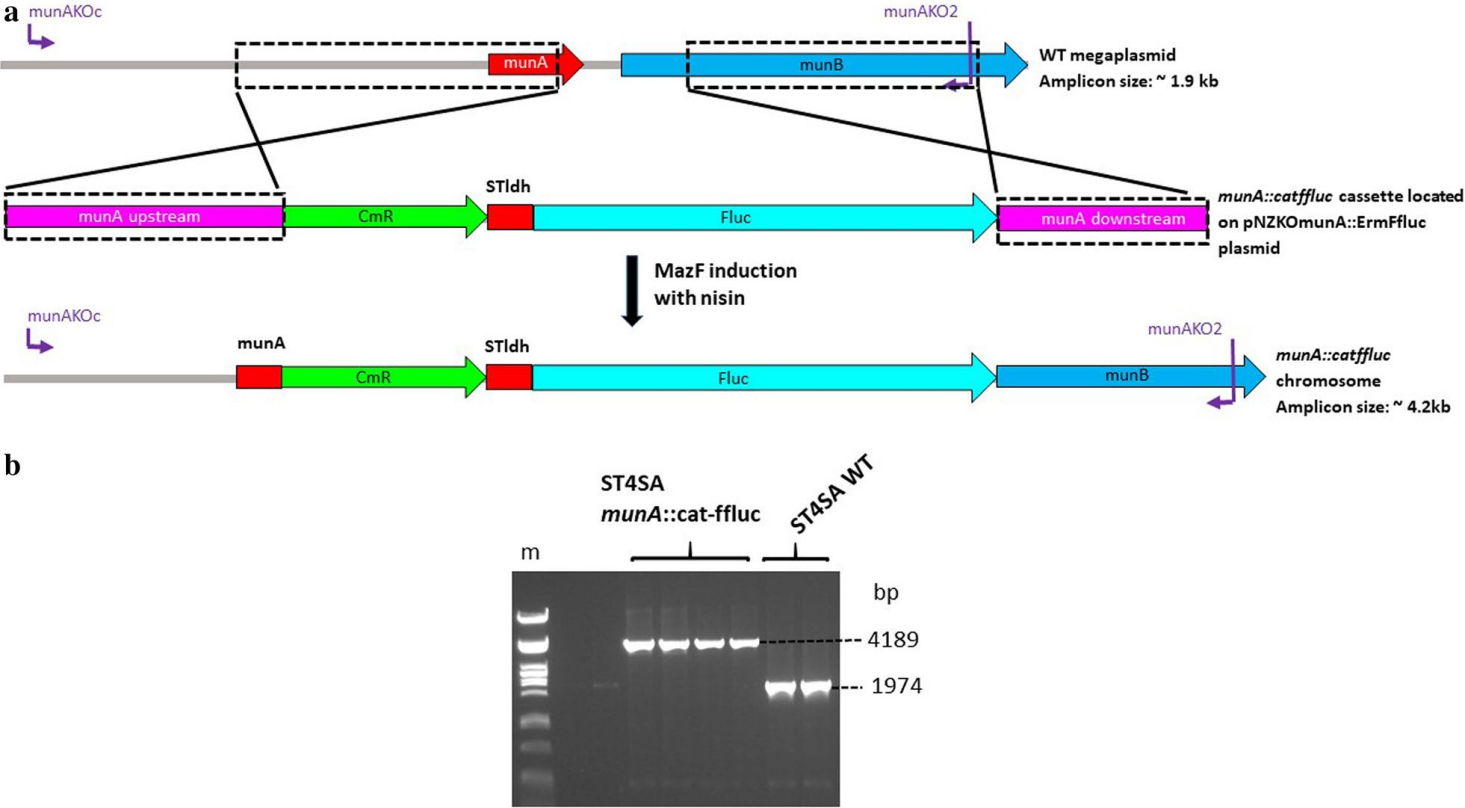
Gene deletion and integration via homologous recombination into the genome of *E. mundtii* ST4SA at the *munA* bacteriocin gene locus to create *E. mundtii* ST4SA *munA*::*cat*-*ffluc.* **a** Homologous recombination between the wild-type (WT) *E. mundtii* ST4SA *munA*-carrying megaplasmid and the *munA::catffluc* cassette. Boxed regions show the upstream (~ 0.9 kb) and downstream regions (~ 0.6) of homology on the megaplasmid and the pNZKOmunACatFfluc knockout (KO) vector. Cells harboring the *munA* KO vector were selected on Cm and Em, followed by nisin induction for MazF toxin expression to select for mutants that have lost the plasmid backbone bearing *erm* and *mazF* genes. Double crossover mutants were selected and screened by PCR using the indicated primer combinations. **b** PCR amplification of WT and *munA* deletion and insertion mutants using the primer pair indicated in panel (**a**). Primer pairs are shown in purple. (m) Lambda DNA digested with *Pst*I (NEB). Amplicons from four *munA* mutant and two WT colonies, respectively, are shown

To demonstrate the feasibility and efficiency of the nisin-MazF counter-selection marker system in LAB, the *E. mundtii* ST4SA *munA* mundticin bacteriocin gene located on a megaplasmid [43] and the *plaA* plantaricin bacteriocin gene located on the *L. plantarum* 423 pPLA4 plasmid [44] were targeted for inactivation. The *munA* knock-out (KO) plasmid carried a 2471 bp *cat*-*ffluc* gene cassette (see Additional file 1: Fig. S3), while the *plaA* KO plasmid carried a 3204 bp *erm*-*ffluc* gene cassette (see Additional file 1: Fig. S4), flanked by regions of homology required for homologous recombination. The pNZKOmunA::CatFfluc included a 135 bp internal fragment of the *mun*A ORF in the upstream region of homology, resulting in the deletion of 18 bp of the 153 bp *munA* gene in all *E. mundtii* ST4SA *munA::cat*-*ffluc* mutants (Fig. 3a). Refer to Table 2 for a detailed description of the components of all regions of homology for targeted gene inactivation. Similarly, pNZKOplaA::ErmFfluc plasmid included a 69 bp internal fragment of the *plaA* ORF in the downstream region of homology, resulting in the deletion of 102 bp of the 171 bp *plaA* gene in all *L. plantarum* 423 *plaA::erm*-*ffluc* mutants (see Additional file 1: Fig. S5). Double crossover mutants were isolated by following the protocol described in “Materials and methods” (also refer to Additional file 1: Fig. S6). *Enterococcus mundtii* ST4SA *munA::cat*-*ffluc* double- crossover mutants retained resistance to Cm, but not Em (erythromycin), in turn indicating the loss of the plasmid backbone DNA encoding the *mazF* and *erm* genes. No double-crossover mutants were obtained in the absence of antibiotic selection. *Lactobacillus plantarum* 423 *plaA::erm*-*ffluc* mutants retained resistance to Em, but not to Cm, indicating that the *erm*-*ffluc* gene cassette was successfully integrated onto the pPLA4 plasmid without the plasmid backbone DNA encoding the *mazF* and *cat* genes. Knockout mutants that appeared on nisin plates were screened by PCR and DNA sequencing using primer combinations listed in Additional file 1: Table S2. *Enterococcus mundtii* ST4SA *munA::cat*-*ffluc* mutants contained the 2471 bp *cat*-*ffluc* “cargo” DNA via allelic exchange, with the consequent deletion of a 260 bp DNA fragment from the *munA* operon harbored on a megaplasmid as confirmed by PCR as well as sequencing (Fig. 3b). Similarly, *L. plantarum* 423 *plaA::erm*-*ffluc* mutants retained the *erm*-*ffluc* cassette via allelic exchange and the deletion of a 208 bp fragment from the *plaA* operon on the pPLA4 native plasmid as confirmed by PCR as well as sequencing (see Additional file 1: Fig. S5).

Supernatants isolated from *L. plantarum* 423 *plaA::erm*-*ffluc* and *E. mundtii* ST4SA *munA::cat*-*ffluc* mutants did not result in the formation of clear inhibition zones on plates overlaid with the sensitive *L. monocytogenes* EGDe strain as compared to the WT derivatives of each strain (see Additional file 1: Fig. S7). Similarly, supernatants isolated from sonicated *plaA*^−^ and *munA*^−^ mutant cell cultures did not result in the formation of inhibition zones on agar plates overlaid with *L. monocytogenes* EGDe (not shown). These results confirmed that the bacteriocin genes of the probiotic strains were successfully inactivated using the nisin-MazF counter-selection method. To test the active expression of the integrated *ffluc* bioluminescence gene, *L. plantarum* 423 *plaA::erm*-*ffluc* and *E. mundtii* ST4SA *munA::cat*-*ffluc* mutants were imaged using the Caliper in vivo imaging system (IVIS; Caliper Life Sciences, Hopkinton, MA, USA). A strong bioluminescent signal was detected from colonies formed on agar plates for each of the mutant strains (see Additional file 1: Fig. S8).

### Integration and removal of the FRT-flanked *erm* gene in *L. plantarum* 423 for marker recycling

**Fig. 4:**
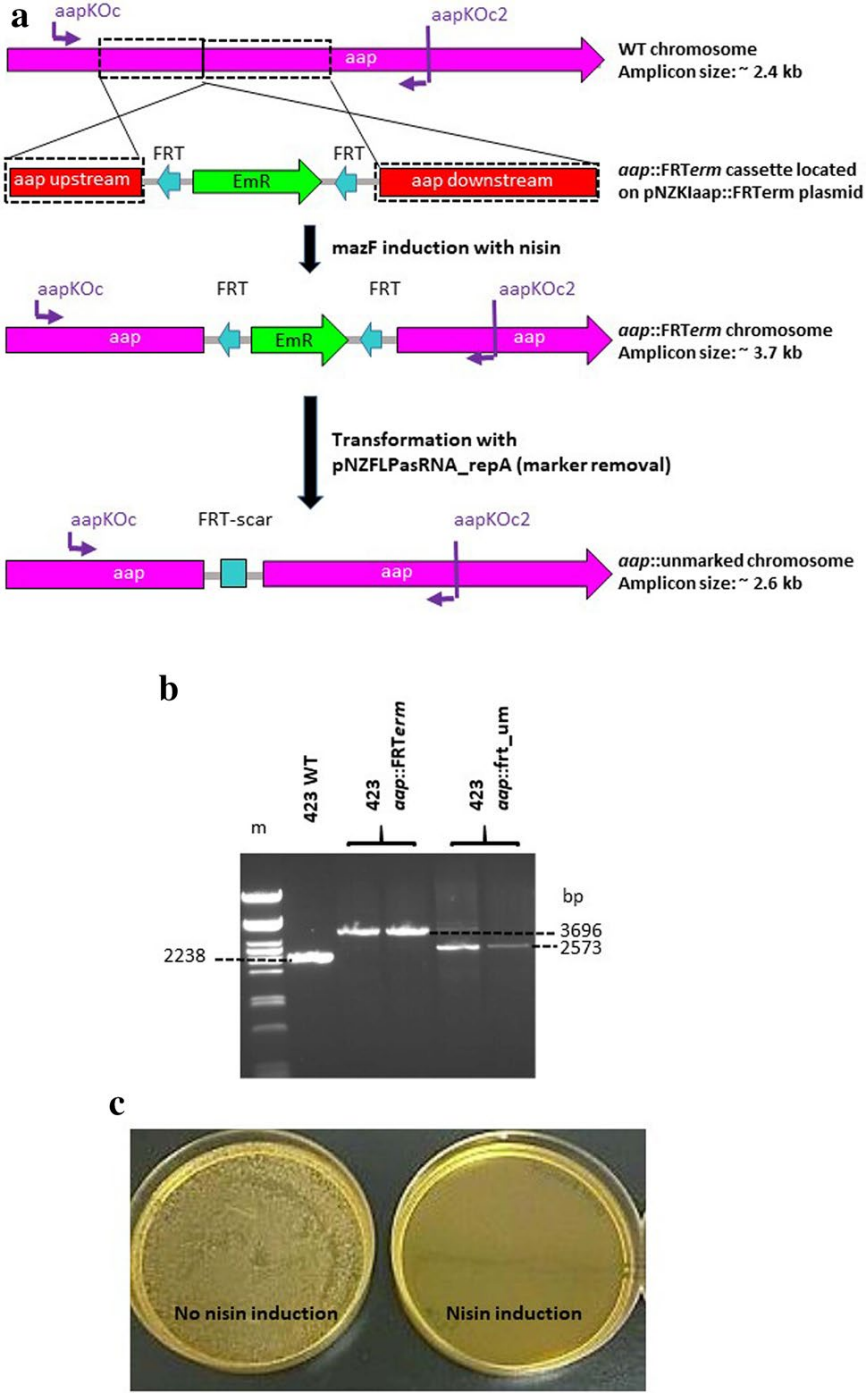
Gene inactivation and integration via homologous recombination into the genome of *L. plantarum* 423 at the *aap* adhesion gene locus to create *L. plantarum* 423 *aap*::FRT*erm* and *L. plantarum* 423 *aap*::frt_um (um-unmarked). **a** Homologous recombination between the wild-type (WT) *L. plantarum* 423 chromosome and the aap::FRT*erm* cassette and selection of unmarked *aap* double-crossover mutants. Boxed regions show the upstream and downstream regions of homology (~ 0.9 kb) on the WT *L. plantarum* 423 chromosome and plasmid pNZKIaap::FRTerm knock-in (KI) vector. Cells harboring the *aap* KI vector were selected on Cm and Em, followed by nisin induction for MazF toxin expression to select for mutants that have lost the plasmid backbone bearing *cat* and *mazF* genes. Double crossover mutants were selected and screened by PCR using the primer combinations indicated in purple. **b** PCR amplification of WT *L. plantarum* 423 and *aap* insertion mutants using the primer pair indicated in panel A. Additionally, the Em resistance marker was recycled via excision by FLP recombinase. (m) Lambda DNA digested with *Pst*I (NEB). Amplicons from one WT, two *aap*::FRT*erm* insertion mutant and two *aap*::unmarked colonies are shown. **c** MRS agar plates showing the effectiveness of the *repA* asRNA induction of FLP recombinase-bearing plasmid loss in the absence of nisin (no nisin induction) and in the presence of nisin (nisin induction). Colonies that have lost the *repA*-bearing plasmid were isolated via replica plating

To overcome the limited availability of antibiotic resistance markers that are suitable for use in LAB as integrative selective markers, the *S. cerevisiae* FLP-FRT recombination system was adapted for marker recycling. A gene disruption mutant of the *L. plantarum* 423 *aap* adhesion-associated gene was generated by utilizing the *erm* gene flanked by two direct repeat FRT recombination targets for excision by FLP recombinase. The generated sizes of the WT *L. plantarum* 423 and integrant loci are shown in Fig. 4a. *Lactobacillus plantarum* 423 *aap::frt*-*erm* mutants were successfully isolated via nisin induction of the *mazF* toxin gene. Double-crossover mutants selected on nisin-supplemented MRS agar plates retained resistance to Em, but not Cm, confirming the loss of the *cat* and *mazF* gene coding plasmid backbone. The recombination event, namely, the insertion of the FRT-*erm* gene onto the *aap* ORF locus, was confirmed by PCR and the resulting amplicon sizes are shown in Fig. 4b. PCR amplification of *aap* mutant gDNA resulted in a 3696 bp fragment compared to the 2238 bp fragment in the WT derivative.

*Lactobacillus plantarum* 423 *aap::frt*-*erm* mutants transformed with the pNZFLPasRNA_repA plasmid, were resistant to Em and Cm (resulting from the *cat* gene on the newly-introduced plasmid). The induction of FLP recombinase resulted in the isolation of unmarked *aap* mutant *L. plantarum aap*::frt_um (um—unmarked) colonies that have lost the *erm* gene via FRT-FLP excision as confirmed by PCR and sequencing (Fig. 4a, b).

Once excision of the *erm* antibiotic resistance marker in *L. plantarum aap*::frt_um colonies was confirmed, loss of the FLP recombinase plasmid was stimulated by the expression of a 350 bp asRNA *repA* transcript by induction with nisin. *Lactobacillus plantarum aap*::frt_um mutants did not show any colony growth on MRS agar plates supplemented with nisin and Cm, indicating plasmid loss, while colonies that were not induced by nisin showed an abundance of growth (indicating the presence of the plasmid, Fig. 4c).

### Isolation of *srtA* and *srtC* deletion mutants using regions of homology containing small (< 60 bp) 5′ and 3′ end sequences

**Fig. 5:**
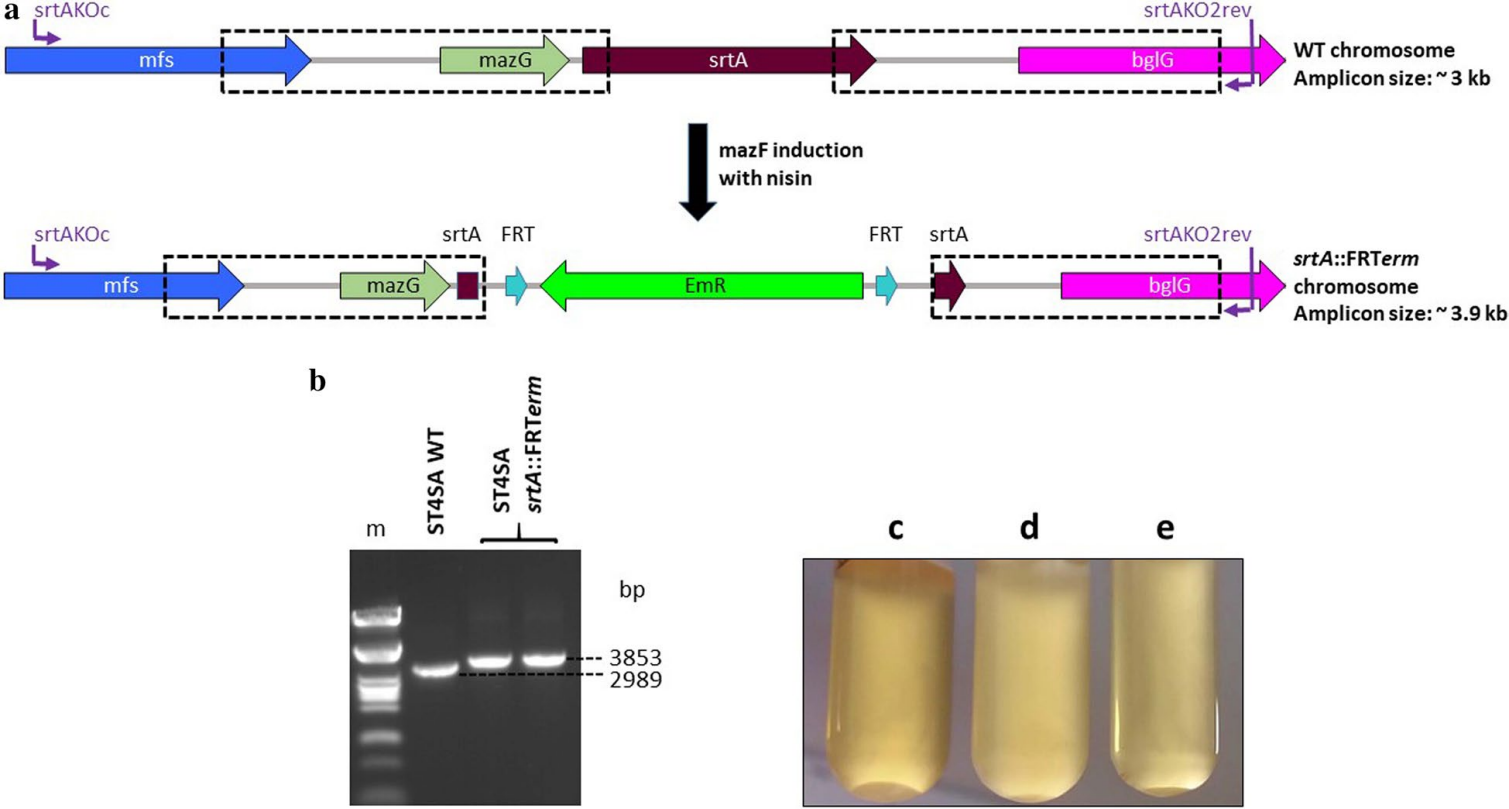
Gene deletion and integration via homologous recombination into the genome of *E. mundtii* ST4SA at the *srtA* locus to create *E. mundtii* ST4SA *srtA*::FRT*erm*, and *E. mundtii* ST4SA *sortase A* aggregation substance (AS) cell clumping assay. **a** Schematic representing the wild-type (WT) *E. mundtii* ST4SA *srtA* gene locus and the recombinant *srtA* deletion and FRT-*erm* integration site. Boxed regions show the upstream and downstream regions of homology (~ 1 kb) on the WT chromosome and the recombinant *srtA*::FRT*erm* locus. Cells harboring the *srtA* knockout vector were selected on Cm and Em, followed by nisin induction for MazF toxin expression to select for mutants that have lost the plasmid backbone bearing *cat* and *mazF* genes. Double crossover mutants were selected and screened by PCR using the primer combinations shown in purple. **b** PCR amplification of WT and *srtA* deletion and insertion mutants using the primer pair indicated in panel A. (m) Lambda DNA digested with *Pst*I (NEB). Amplicons from one WT and two *srtA*::FRT*erm* insertion mutant colonies are shown. **c** MRS broth with the WT strain containing SrtA AS. **d** MRS broth containing the *E. mundtii* ST4SA *srtA*::FRT*erm* deletion mutant strain lacking SrtA AS. **e** MRS broth with *E. mundtii* ST4SA *srtC*::FRT*erm* deletion mutant strain containing SrtA AS

Although the PnisA-MazF counter-selection system proved to be functional, the *E. mundtii* ST4SA *srtA* and *srtC* genes did not contain sufficient restriction enzyme sites to allow cloning of integrative gene cassettes flanked by homologous arms. A strategy to introduce new restriction sites would facilitate the cloning of integrative genes in between homologous regions to enable the inactivation of target sequences with insufficient restriction enzyme digestion sites. To do this, *srtA* and *srtC* KO plasmids carrying upstream and downstream regions of homology with new restriction sites for integrative gene cassette insertion were constructed (see Additional file 1: Figs. S9 and S10). Both KO plasmids were designed to facilitate the deletion of the majority of the *srtA* and *srtC* genes sequences (with < 60 bp of the 5′ and 3′ ends of the gene coding sequences included in the upstream and downstream regions of homology). The FRT-flanked *erm* gene was utilized as integrative cassette and the anticipated sizes of the WT *E. mundtii* ST4SA and integrant *srtA* loci are shown in Fig. 5a. *Enterococcus mundtii* ST4SA WT and integrant *srtC* loci are shown in Additional file 1: Fig. S11. The deletion mutant isolation protocol was followed to enrich for recombinant cells that have undergone the desired double-crossover recombination event. *Enterococcus mundtii* ST4SA *srtA::frt*-*erm* mutants were successfully isolated that resulted in the deletion of a 580 bp fragment of the 714 bp *srtA* ORF as confirmed by PCR and sequencing (Fig. 5b). Similarly, the expected band sizes of *E. mundtii* ST4SA *srtC::frt*-*erm* mutants were obtained as confirmed by PCR, sequencing and restriction digests, that resulted in the deletion of a 726 bp internal fragment of the 826 bp *srtC* ORF (see Additional file 1: Fig. S11).

To verify the loss of the *E. mundtii* ST4SA *srtA* aggregation substance’s (AS) ability in *srtA* KO mutants to attach secreted adhesion proteins to the bacterial cell surface, a cell clumping assay was performed. In WT *E. mundtii* ST4SA cells, AS expression lead to a marked clumping of bacterial cells to form large cell aggregates that settled at the bottom of the tube (Fig. 5c). In the *srtA* KO strain lacking AS, the cell suspension remained turbid, thus indicating the loss of the cells’ ability to form aggregates compared to the WT (Fig. 5d). In the tube containing *E. mundtii* ST4SA *sortaseC* KO cells, the bacteria maintained the ability to form aggregates that settled at the bottom of the tube (Fig. 5e). These results confirmed the SortaseA mutant phenotype and that the SortaseA AS is required for the aggregation of cells.

## Discussion

The functional genetic analysis of genes that confer specific phenotypical properties in LAB is highly dependent on the application of an effective counter-selection marker system for the easy and efficient isolation of chromosomally- or plasmid-located allelic exchange mutants [11, 26, 27]. While the use of replicative plasmids to study protein functions is easy to implement, multi-copy plasmids are not suited for in vitro or in vivo models where antibiotic selection for plasmid maintenance is not possible [12]. The integration or deletion of genes at any specified locus circumvents antibiotic selection related issues. Most of the techniques currently used for the isolation of homologous recombination mutants in LAB are subject to the following limitations: a lack of broad applicability amongst different LAB species, an inability to target specific gene loci, a shortage of suitable antibiotic resistance markers, and failure to isolate irreversible double-crossover mutants [17–19, 28, 29, 45, 46].

Counter-selection markers are invaluable for the construction of stable double-crossover mutants, especially in probiotic LAB, where the mechanisms by which they exert their beneficial effects on the consumer can be studied by reverse genetic analysis [26, 46]. Nevertheless, the identification and optimization of suitable counter-selection markers is a challenging and laborious task.

In this study, a method was developed to easily and efficiently isolate stable irreversible double-crossover mutants in *L. plantarum* 423 and *E. mundtii* ST4SA. The *E. coli mazF* toxin gene was placed under the control of the PnisA nisin-inducible promoter that is strictly associated with the *nisR* and *nisK* nisin signal regulatory genes, to form the pNZmazFnisRK destination plasmid [37]. A major advantage of the NICE system over other inducible gene expression systems, is the tightly controlled gene expression that has been used to produce large amounts of enzymes for food, medical or technical applications [47, 48]. This study has shown that MazF expression is tightly controlled by the nisin-inducible promoter. This eliminates any potential MazF-induced premature cell growth arrest as a result of promoter leakiness.

Growth conditions were successfully optimized for the *mazF* counter-selection gene to promote the death of cells harbouring *mazF* upon induction of the PnisA promoter with nisin. This approach ensures that transformants that have undergone only a single event of homologous recombination (thus retaining a copy of the plasmid-located counter-selection marker in the chromosome), are eliminated in the presence of nisin-induced MazF toxin. This method was used to construct deletions within the ORFs of specific genes, to introduce a gene of interest, to construct an unmarked mutation and to realize gene deletion mutants using small regions of homology. In the first case, bacteriocin gene deletion mutants of *L. plantarum* 423 and *E. mundtii* ST4SA were constructed, while simultaneously inserting the large 1.6 kb *ffluc* firefly luciferase reporter gene. The *L. plantarum* 423 *plaA* bacteriocin KO plasmid was designed to delete the majority of the *plaA* ORF while ensuring that none of the adjacent genes would be affected. In a previous study [49], the *L. plantarum* 423 WT strain was cured of the pPLA4 *plaA* gene-encoding plasmid to produce a bacteriocin-deficient mutant. While their approach proved successful, the loss of the whole plasmid may be undesirable because of the consequent loss of all bacteriocin-adjacent genes. The *E. mundtii* ST4SA *munA* bacteriocin KO plasmid was designed for the deletion of 18 bp of the *munA* ORF. The bacteriocin gene deletions of both *L. plantarum* 423 and *E. mundtii* ST4SA resulted in the loss of bacteriocin production, confirmed against *L. monocytogenes* EGDe. Supernatants isolated from the bacteriocin-deficient mutant strains lacked the ability to form clear inhibition zones as compared to the wild type derivatives. Bioluminescence imaging revealed that the integrated *ffluc* gene was actively expressed in each of the bacteriocin KO mutants. Strong bioluminescence emission was detected from colonies on MRS agar plates, indicating the functional expression of the Ffluc protein at the integrated loci. These results demonstrate the feasibility and applied utility of the newly developed counter-selectable marker system.

Desired double-crossover mutants are usually isolated using an antibiotic resistance gene as integrative selective marker [17]. However, repeated manipulations of any bacterial chromosome can only be achieved by the generation of markerless integration mutants due to a limited availability of suitable antibiotic resistance markers. An easy-to-implement marker recycling system for LAB allows microbiologists to genetically engineer LAB strains for biotechnological production processes. In this study, the *S. cerevisiae* FLP-FRT recombination system was modified for application in LAB. The FLP-FRT recombination system is present in most yeast strains and is encoded by the 2 µm (6.4 kb) plasmid [50, 51]. Two flanking 34 bp FLP recognition targets (FRT) are necessary for successful excision of an integrated fragment by FLP recombinase. The yeast FLP-FRT system has been successfully applied in several pathogenic and non-pathogenic Gram-negative and Gram-positive bacteria [36, 52–54]. The *L. plantarum* 423 *aap* mucus adhesion gene was targeted for inactivation with the insertion of an FRT-flanked *erm* resistance gene [55]. Once FRT-*erm* gene insertion was confirmed, a two-step process was followed for marker recycling. In the first step, FLP recombinase activity was induced by transformation with plasmid pNZFLPasRNA_repA to produce unmarked *aap* KO mutants. Secondly, once excision of the *erm* gene was confirmed, loss of the pNZFLPasRNA_repA plasmid was stimulated by a 350 bp *repA* antisense RNA (asRNA) transcript placed under the control of the PnisA nisin-inducible promoter. The *repA* asRNA is a single-stranded RNA molecule that is complementary to the *repA* mRNA molecule of pNZ8048-derived vectors. The *repA* asRNA inhibits translation of the r*epA* origin of replication (ori) by binding to the first 350 bp of the complementary *repA* mRNA transcript [56]. As a result, the strain was cured of the *repA* ori-containing plasmid via *repA* protein synthesis inhibition. Previous studies have used asRNA successfully to modulate the expression of specific genes or to prevent the proliferation of bacteriophages in Gram-positive bacteria, including many LAB sp. [57–63]. The system developed in this study requires minimal effort (days) for the isolation of unmarked mutants based on the strength and specificity of the NICE system. The resulting *aap* KO unmarked strain, free of antibiotic selection markers, may be used for further genetic manipulations of the *L. plantarum* 423 chromosome in a similar manner.

To further demonstrate the strength of the newly developed system, gene deletion mutants of the *E. mundtii* ST4SA *srtA* and *srtC* genes were generated using homologous arms containing < 60 bp regions (5′ and 3′) homologous to the target genes. This approach ensured that none of the adjacent genes were affected while deleting the majority of each target. Emphasis is placed on the introduction of new restriction sites in homologous arm regions for the insertion of integration cassettes to ensure optimal cloning of integrative cassettes. This strategy ensures the inactivation of gene targets that may not contain sufficient restriction sites to facilitate the cloning of integrative genes, usually antibiotic resistance genes, flanked by homologous regions. The technique used in this study ensures the isolation of recombination mutants even when using small regions (< 60 bp) of the coding sequence of a target, included in larger homologous arms containing sequences adjacent to the target genes.

## Conclusions

The method described in this paper is easy to implement, highly efficient and can be used to manipulate the chromosomes of *Lactobacillus* and *Enterococcus* spp. Furthermore, the strategy is well adapted for use in other LAB sp. due to the broad applicability of the nisin expression system. This provides a unique opportunity to study the role of specific probiotic LAB genes in complex environments using reverse genetics analysis. In addition to the efficient deletion or integration of genes at any defined loci, the use of the FLP/FRT recombination system provides marker recycling for further manipulations of LAB strains.

## Materials and methods

### Bacterial strains, plasmids and culture media

Bacterial strains and general cloning plasmids are listed in Table 1 and Additional file 1: Table S1. All subcloning experiments were done in *E. coli* DH5α [64] and *E. coli* MC1061 (Mobitec, Göttingen, Germany). *Escherichia coli* strains were grown in Luria–Bertani (LB), brain heart infusion (BHI) broth or solid agar (1.5% w/v) (Biolab Diagnostics, Midrand, South Africa) and incubated at 37 °C with rotary shaking at 200 rpm. Forty micrograms per milliliter of 5-bromo-4-chloro-3-indolyl β-d-galactopyranoside (X-gal) was added to *E. coli* LB growth media when required for blue-white colony screening. The probiotic LAB strains *L. plantarum* 423 and *E. mundtii* ST4SA were grown as static cultures at 30 °C in MRS broth (Biolab Diagnostics) or on MRS agar plates. *Lactococcus lactis* pNZ9000 was grown at 30 °C in M17 broth (Biolab Diagnostics) without shaking, or on agar plates supplemented with 0.5% (w/v) glucose. *Escherichia coli* strains containing plasmids (Table 1) were cultured in LB or BHI medium supplemented with either 200 µg/ml Em, 10 µg/ml Cm or 100 µg/ml ampicillin (Amp). Recombinant LAB strains containing plasmids, and integrative mutants (Table 2), were cultured in MRS or M17 medium supplemented with 10 µg/ml Em or 10 µg/ml Cm for *L. plantarum* 423, and 5 µg/ml Em or 5 µg/ml Cm for *E. mundtii* ST4SA and *Lc. lactis* pNZ9000. *Listeria monocytogenes* EGDe was grown in BHI media supplemented with 7.5 µg/ml Cm and incubated at 37 °C on an orbital shaker (200 rpm).

**Table 1:** Bacterial strains and plasmids used in this study

Strain or plasmid	Description	Reference or source
Strains		
*E. coli*		
DH5α	Host strain used for general subcloning	[61]
MC1061	Host strain used for subcloning with *Lc. lactis* derived pNZ8048 vector; *recA* positive strain	Mobitec, Göttingen, Germany
*Lactobacillus plantarum*		
423	Probiotic with multiple adhesion genes and a plantaricin bacteriocin producer; forms part of the entiro™ probiotic; originally isolated from sorghum beer	Cipla Medpro (Pty.) Ltd.
423 pNZmazFnisRK	Contains the pNZmazFnisRK plasmid; Cm^R^	This study
*Enterococcus mundtii*		
ST4SA	Probiotic with multiple adhesion genes and a mundticin bacteriocin producer; forms part of the entiro™ probiotic; originally isolated from soybeans	Cipla Medpro (Pty.) Ltd.
ST4SA pNZmazFnisRK	Contains the pNZmazFnisRK plasmid; Cm^R^	This study
*Lactococcus lactis*		
pNZ9000	Standard host strain for nisin regulated gene expression; harbors the *nisR* and *nisK* nisin regulatory genes integrated into the *pepN* gene locus	Mobitec, Göttingen, Germany
pNZ9000 pNZmazF	Contains the pNZmazF plasmid; Cm^R^	This study
Plasmids		
pNZ8048	Broad-host range vector; *E. coli* shuttle vector; LAB expression vector containing nisin A inducible-promoter (PnisA); Cm^R^	Mobitec, Göttingen, Germany; [32]
pGKV223D	*E. coli/*LAB shuttle vector; LAB expression vector with L23 promoter; Em^R^	University of Gröningen, The Netherlands
pNZnisRK	pNZ8048 vector carrying the *nisR* and *nisK* regulatory genes for cloning in LAB strains that do not have the regulatory genes integrated onto the chromosome; Cm^R^	This study
pNZmazF	pNZ8048 vector carrying the *E. coli mazF* toxin gene under the control of the PnisA promoter; Cm^R^	This study
pNZmazFnisRK	pNZnisRK vector carrying the *E. coli mazF* toxin gene under the control of the PnisA promoter; Cm^R^	This study
pNZmazFnisRKerm	pNZ8048nisRK vector carrying the *E. coli* mazF toxin gene under the control of the PnisA promoter and the *erm* gene; Cm^R^, Em^R^	This study
pBluescriptKS	PCR cloning vector; Amp^R^	Stratagene, California, USA.
pKSFRT	pBluescriptKS plasmid carrying two *flippase* (FLP) recombination target sequences (FRT) in a direct repeat orientation; Amp^R^	This study
pKSFRTErm	pBluesriptKS plasmid carrying the *erm* gene flanked by two FRT sequences in a direct repeat orientation; Em^R^, Amp^R^	This study
pGKVPldhFLP	pGKV223D vector carrying the *flippase* (FLP) gene downstream of the constitutive *L. plantarum* 423 *lactate dehydrogenase* gene Pldh promoter; Em^R^	This study
pNZasRNA_repAnisRK	pNZmazFnisRK vector carrying a 350 bp asRNA_repA transcript downstream of the nisin-inducible PnisA promoter; Cm^R^	This study
pNZasFLPasRNA_repA	pNZasRNA_repAnisRK vector carrying the flippase (FLP) gene downstream of the constitutive *L. plantarum* 423 *lactate dehydrogenase* gene Pldh promoter; Cm^R^	This study

Cm^R^: chloramphenicol resistance; Em^R^: erythromycin resistance; Amp^R^: ampicillin resistance

**Table 2:** List of integration vectors and recombinant LAB strains

Plasmid	Modified strain	Locus	Upstream region of homology	Downstream region of homology	Region deleted on chromosome/plasmid	Element(s) integrated onto chromosome/plasmid
pNZKOplaA::ErmFfluc	*L. plantarum* 423 plaA::erm-ffluc	plaA	902 bp upstream of plaA	69 bp internal fragment of plaA, 330 bp plaB bacteriocin immunity gene and 214 bp of plaC bacteriocin translocation protein	208 bp, including 102 bp of plaA ORF	3204 bp erm-ffluc gene cassette
pNZKOmunA::CatFfluc	*E. mundtii* ST4SA munA::cat-ffluc	munA	911 bp upstream of munA, including 135 bp internal fragment of munA ORF	633 bp internal fragment of munB translocation gene	260 bp, including 18 bp of munA ORF	2471 bp cat-ffluc gene cassette
pNZKIaap::FRTerm	*L. plantarum* 423 aap::frt-erm	aap	909 bp internal fragment of aap ORF	929 bp internal fragment of aap ORF	None	1458 bp FRT-flanked erm gene
pNZKIaap::FRTerm	*L. plantarum* 423 aap::frt_um		909 bp internal fragment of aap ORF	929 bp internal fragment of aap ORF	1063 bp FRT-flanked erm resistance gene excised	335 bp including a 96 bp FRT-scar
pNZKOsrtA::FRTerm	*E. mundtii* ST4SA srtA::frt-erm	srtA	1011 bp upstream of srtA, including 57 bp internal fragment of srtA ORF, 333 bp mazG nucleotide pyrophosphohydrolase gene and 270 bp fragment of mfs transporter ORF	1011 bp downstream of srtA, including 57 bp internal fragment of srtA ORF and 576 bp internal fragment of bglG transcriptional anti-terminator ORF	580 bp internal fragment of the srtA ORF	1458 bp FRT-flanked erm gene
pNZKOsrtC::FRTerm	*E. mundtii* ST4SA srtC::frt-erm	srtC	1011 bp upstream of srtC, including 59 bp of srtC ORF and 892 bp of EbpCfm pilus subunit protein gene	376 bp downstream of srtA, including 60 bp of srtC ORF and 225 bp internal fragment of hemolysin III ORF	726 bp internal fragment of the srtC ORF	1458 bp FRT-flanked erm gene

The integration vectors and their relevant characteristics are shown, and their construction is described in detail in the main text (pNZKOmunA::CatFfluc, pNZKIaap::FRTerm and pNZKOsrtA::FRTerm), Additional file 1: Text S1 (pNZKOplaA::ErmFfluc and pNZKOsrtC::FRTerm) and Additional file 1: Figs S2–S6. *plaA*: plantaricin 423 bacteriocin gene; *erm*: erythromycin resistance gene; *ffluc*; *Photinus pyralis* firefly luciferase gene; *munA*; mundticin ST bacteriocin gene; *cat*: chloramphenicol resistance gene; *aap*; FRT: flippase recombination target; *L. plantarum* 423 adhesion gene; um: unmarked; *srtA*: *E. mundtii* ST4SA *sortaseA* gene; *srtC*: *E. mundtii* ST4SA *sortaseC* gene

### DNA manipulation procedures and transformation

Nucleic acid manipulations and general cloning procedures were carried out according to standard protocols, as described by Sambrook and Russel [65]. DNA restriction and modification enzymes were purchased from New England Biolabs (NEB, Ipswich, MA, USA) and were used as recommended by the manufacturer. Oligonucleotides were purchased from Inqaba Biotechnical Industries (Pretoria, South Africa). PCR amplifications were performed using Q5 high-fidelity PCR DNA polymerase (NEB) in a SwiftMinipro thermal cycler (Esco Healthcare, Malaysia). DNA fragments were purified from agarose gels using the Zymoclean™ gel DNA recovery kit (Zymo Research Corporation, Irvine, CA, USA). *Escherichia coli* plasmid DNA was purified using the PureYield™ plasmid miniprep system (Promega, Madison, WI, USA). Genomic DNA (gDNA) of *E. coli* and LAB strains was purified using the ZR Fungal/Bacterial DNA miniprep kit (Zymo Research Corporation) following the manufacturer’s instructions. Electrotransformation of *E. coli*, *L. plantarum* 423 and *E. mundtii* ST4SA was achieved as described previously [25], using the Bio-Rad Gene Pulser electroporation system (Bio-Rad Laboratories, Hercules, CA, USA). *Lactococcus lactis* NZ9000 was electroporated as suggested by the supplier, using standard procedures (Mobitec).

### Construction of plasmids

The integration vectors for use in *L. plantarum* 423 and *E. mundtii* ST4SA were based on the pNZ8048 *Lc. lactis* NICE system plasmid (Mobitec), containing the PnisA *nisA* gene promoter region, a multiple cloning site (MCS), *E. coli*/*Lc. lactis repC* and *repA* replication genes for replication in LAB and *E. coli*, the *cat* gene for Cm resistance and the termination (T) sequence of the *Lc. lactis pepN* gene. DNA primers used are listed in Additional file 1: Table S2. The *nisK* and *nisR* regulatory genes were amplified from *Lc. lactis* pNZ9000 gDNA, using primers nisRK1 and nisRK2. The 2162 bp amplicon was digested with *Hin*dIII and *Xho*I and cloned into pNZ8048 after digestion of the vector with the same restriction enzymes, yielding plasmid pNZnisRK. A schematic diagram summarizing the construction of pNZmazF and pNZmazFnisRK is shown in Additional file 1: Fig. S12. The pNZmazF and pNZmazFnisRK plasmids were constructed to test the functionality of the nisin-inducible promoter in *Lc. lactis* pNZ9000, *L. plantarum* 423 and *E. mundtii* ST4SA. The *mazF* toxin gene was amplified from genomic DNA isolated from *E. coli* DH5α, using primers mazF1 and mazF2. The generated 342 bp amplicon was cloned into the MCSs of pNZ8048 and pNZnisRK, using *Nco*I and *Hin*dIII, to yield pNZmazF and pNZmazFnisRK, respectively. The integration vectors and their relevant characteristics are listed in Table 2.

The *E. mundtii* ST4SA *munA* bacteriocin gene knock-out (KO) plasmid was constructed as follows. First, a complete 1809 bp region of homology that included the *munA* ORF was obtained by PCR using the primer pair munAKO1/munAKO2, incorporating *Eco*RI and *Xba*I digestion sites (see Additional file 1: Fig. S2). The generated amplicon was digested with *Eco*RI/*Pvu*II and *Hpa*I/*Xba*I, resulting in a *munA* 911 bp upstream- and a 633 bp downstream-region of homology, respectively, and the removal of 18 bp from the *munA* ORF. Next, the two regions of homology were joined together with a blunt-ended 2480 bp *cat*-*ffluc* gene cassette generated via PCR amplification using primer pair cat1/fluc2 and plasmid pGKVCatFflucST4SA (Additional file 1: Table S1) as source DNA. The *cat*-*ffluc* gene cassette contained the *cat* gene for chloramphenicol resistance and the firefly luciferase gene (*ffluc*) from *Photinus pyralis* fused to the strong constitutive *E. mundtii ldh* gene (Pstldh) promoter. This *cat*-*ffluc*-interrupted *munA* region was then cloned into the *Eco*RI/*Xba*I double-digested pBluescriptKS cloning vector, yielding plasmid pKSmunA::CatFfluc. Finally, the *cat*-*ffluc* gene cassette flanked by the *munA* upstream/downstream regions of homology (4024 bp) was PCR amplified from pKSmunA::CatFfluc and cloned into the *Stu*I-linearized pNZmazFnisRKerm plasmid, containing both *cat* and *erm* antibiotic resistance genes, yielding plasmid pNZKOmunA::CatFfluc.

The *L. plantarum* 423 *aap* adhesion gene knock-in (KI) plasmid was constructed by PCR, amplifying a 1848 bp internal fragment of the *aap* ORF using primer pair aapKO1/aapKO2 (see Additional file 1: Fig. S13). The resulting amplicon was triple-digested with *Eco*RI/*Hpa*I/*Xba*I (*Hpa*I has a single cut site approximately in the middle of the *aap* fragment), and ligated to the blunt-ended *flippase* (FLP) recombination target (FRT)—flanked *erm* gene PCR fragment as well as the pBluescriptKS plasmid double-digested with *Eco*RI/*Xba*I, to yield plasmid pKSaap::FRTerm. The 1458 bp FRT-flanked e*rm* gene was generated using primer pair M13for/M13rev and plasmid pKSFRTerm as source DNA (see Additional file 1: Fig. S14). The FRT-*erm* gene flanked by the *aap* upstream/downstream regions of homology (3305 bp) was PCR amplified from pKSaap::FRTerm using primer pair aapKO1/aapKO2 and cloned into the blunt-ended (*Bgl*II, blunted) destination plasmid pNZmazFnisRK to yield plasmid pNZKIaap::FRTerm.

The *E. mundtii* ST4SA *sortase A* (*srtA*) deletion plasmid was constructed by amplifying two regions of homology flanking the *srtA* gene, using primer pairs, srtAKO1for/srtAKO1rev and srtAKO2for/srtAKO2rev. The generated amplicons (~ 1 kb each) were designed to include small internal (< 60-bp) fragments of the *srtA* ORF in both the upstream and downstream regions of homology (see Additional file 1: Fig. S9). The upstream and downstream regions were then digested with *Hin*dIII/*Hpa*I and *Hpa*I/*Xba*I, respectively, ligated to the blunt-ended FRT-*erm* gene PCR fragment and pBluescriptKS digested with *Hin*dIII and *Xba*I, yielding plasmid pKSsrtA::FRTerm. The complete *srtA* region of homology including the FRT-*erm* gene was then PCR amplified from pKSsrtA::FRTerm using primer pair srtAKO1for/srtAKO2rev, and cloned into the *Hin*dIII-linearized and blunt-ended plasmid pNZmazFnisRK, yielding plasmid pNZKOsrtA::FRTerm. Construction of integration plasmids pNZKOplaA::ErmFfluc and pNZKOsrtC::FRTerm is described in the Additional file 1: Text S1 and shown in Additional file 1: Figs. S4 and S10.

Plasmids pKSFRTErm and pNZFLPasRNA_repA were constructed for utilization of the FLP/FRT recombination system of *Saccharomyces cerevisiae* for the generation of unmarked LAB KO mutants and antibiotic marker recycling, as follows (Additional file 1: Figs. S14 and S15). The pKSFRTErm plasmid was constructed by PCR amplification of two 48 bp FRT target sequences using primer pairs FRTfor1/FRTrev1, FRTfor2/FRTrev2 and the *S. cerevisiae* 2 µm (~ 6.4 kb) plasmid as template DNA. The two generated amplicons containing the FRT target sequences were then double-digested with *Hin*dIII/*Sal*I and *Bam*HI/*Pst*I, respectively, and cloned into the pBluescriptKS plasmid in a direct repeat orientation. The resulting plasmid (pKSFRT) was then digested with *Eco*RI (located in-between the two FRT repeats) and ligated to an *erm* gene PCR fragment (erm1/erm2 primer pair) digested with the same enzyme to yield plasmid pKSFRTErm (see Additional file 1: Fig. S14).

The pNZFLPasRNA_repA plasmid was constructed as follows. The 1284 bp *flippase* (FLP) gene was amplified from the 2 µm plasmid using primer pair FLPfor/FLPrev, incorporating the *Nco*I/*Sal*I digestion sites, and ligated to the *Eco*RI/*Nco*I double-digested Pldh promoter and double-digested *Eco*RI/*Sal*I pGKV223D plasmid, yielding plasmid pGKVPldhFLP (see Additional file 1: Fig. S15). Next, a 350 bp *repA* antisense RNA (asRNA) PCR fragment was generated, using primers asRNAfor and asRNArev, and was fused upstream of the nisin-inducible promoter by digestion of the amplicon with *Nco*I and *Hin*dIII and cloned to the pNZmazFnisRK plasmid after digestion with the same restriction enzymes, creating pNZasRNA_repAnisRK. The asRNA_repA fragment was designed for inactivation of the RepA replication protein by binding to the first 350 bp of the *repA* transcript as a reverse-and-complement RNA strand thus inhibiting its translation. Finally, the 1794 bp Pldh-FLP PCR fragment was amplified from plasmid pGKVPldhFLP using primer pair Pldh1/FLPrev and subsequently cloned into the *Bgl*II blunt-ended pNZasRNA_repAnisRK plasmid, yielding plasmid pNZFLPasRNA_repA (see Additional file 1: Fig. S15). The integrity of all plasmids constructed was verified by restriction digests and by PCR using appropriate primer combinations (Additional file 1: Table S2).

### Optimization of MazF toxin expression using the nisin-inducible promoter in LAB

Control *L. plantarum* 423 and *E. mundtii* ST4SA strains transformed with empty pNZ8048 plasmid and recombinant *L. plantarum* 423 and *E. mundtii* ST4SA transformed with the pNZmazFnisRK plasmid were grown in MRS broth for 12 h. One millilitre of the 12 h old cultures was used to inoculate 50 ml of pre-warmed MRS broth. The 50 ml cultures were supplemented with 0 ng/ml, 100 ng/ml, 200 ng/ml, 300 ng/ml, 400 ng/ml and 600 ng/ml nisin (Sigma-Aldrich, St. Louis, MI, USA), respectively, and incubated at 30 °C for 7 h. Every 30 min the optical density (OD_550nm_) reading for each 50 ml culture was measured. All experiments were done with three repeats. Expression of the *mCherry* reporter gene in *Lc. lactis* pNZ9000, *L. plantarum* 423 and *E. mundtii* ST4SA, placed under control of the PnisA nisin-inducible promoter was achieved as described in Additional file 1: Text S1. Agar plates streaked with fluorescing LAB cells were imaged using the IVIS.

### Confirmation of bacteriocin gene knockouts

To confirm bacteriocin gene KO integration mutants of strains *L. plantarum* 423 and *E. mundtii* ST4SA an overlay lawn assay was performed as described by Van Zyl et al. [25] with the following modifications. The *L. monocytogenes* EGDe bacteriocin sensitive strain was grown for 12 h and 100 µl plated onto a BHI agar plate. The cell-free supernatants (adjusted to pH 7.0 with NaOH) of actively growing (12 h) bacteriocin KO strains were collected by centrifugation (8000×*g* for 5 min). The supernatants were sterilized by passage through a 0.22-µm pore size filter using a 5 ml syringe and 30 µl spotted into wells on BHI agar overlaid with *L. monocytogenes* EGDe. This was followed by incubation at 37 °C for 24 h. As positive controls, supernatant from WT strains was used. Additionally, bacteriocin KO strains were sonicated for 3 min using an Omni-Ruptor 400 (Omni International, Kennesaw, GA, USA) for release of intracellular proteins. Briefly, bacteriocin KO cultures were grown for 12 h in 100 MRS broth. Cells were harvested at 13,000×*g* for 10 min, resuspended in 30 ml 10 mM Tris buffer supplemented with 500 mM NaCl and 10 mg/ml lyzozyme, and incubated at 37 °C for 2 h. After incubation, the cells were sonicated for 3 min followed by centrifugation (13,000×*g* for 5 min) and collection of the cell free supernatants. The supernatants containing intracellular proteins were then spotted onto BHI agar plates spread with *L. monocytogenes* EGDe as described before for visualization of inhibition zones. Expression of bioluminescence by *L. plantarum* 423 and *E. mundtii* ST4SA bacteriocin-negative strains was detected as described in Additional file 1: Text S1.

### Clumping response protocol

The bacterial clumping protocol for confirmation of the *E. mundtii* ST4SA *srtA* gene KO strain was adapted from previous studies [66, 67] with the following modifications. A pheromone-containing supernatant was collected by centrifugation (13,000×*g* for 5 min) of a 12-h old WT *E. mundtii* ST4SA strain, followed by filter sterilization through a 0.22-µm-pore size cellulose nitrate filter using a 5 ml syringe. Wild-type and *srtA* KO mutant strains were grown for 12 h in MRS broth and diluted to an OD_600nm_ of ~ 0.06 in 5 ml MRS broth supplemented with 250 µl sterile supernatant from WT *E. mundtii* ST4SA. The cultures were incubated for 12 h at 30 °C without shaking before visualization of clumping.

### Confirmation of successful chromosomal or plasmid integrations

To confirm successful double-crossover recombination events in LAB gene deletion or integration mutants, gDNA was isolated for PCR amplification using the appropriate primer combinations listed in Additional file 1: Table S2. All LAB KO or KI mutants were confirmed by DNA sequencing of PCR products performed by the Central DNA Sequencing Facility of Stellenbosch University.

### Protocol for isolating double-crossover mutants

The complete step-by-step design and timeline for this method of quick and easy isolation of double-crossover KO or KI mutants is shown in Additional file 1: Fig. S6. Upon completion of the construction and transformation of an appropriate integration plasmid in LAB strains, transformants were enriched for by either direct inoculation into MRS broth or by plating onto MRS agar plates, supplemented with both Cm and Em. After an incubation period of 2–3 days at 30 °C, cells grown in broth or colonies observed on agar plates were streaked on fresh agar plates and re-incubated for 24 h at 30 °C until single colonies were observed. Cells carrying the integration plasmid were then inoculated into fresh MRS broth supplemented with Cm and Em, followed by further incubation at 30 °C for 12 h. Two-hundred microliters of the cell suspension was then inoculated into fresh MRS broth supplemented with the antibiotic present in the integrative gene cassette (either Cm or Em) and the appropriate concentration of nisin for MazF toxin expression, followed by incubation at 30 °C for 6 h. After 6 h of incubation and growth recorded at OD_600 nm_, the cell suspensions were serially diluted, and plated onto MRS agar plates supplemented with the appropriate antibiotic and nisin as described above. The plates were incubated for 24 to 48 h at 30 °C or until colonies were observed. Double-crossover recombined mutants appearing on the antibiotic-nisin plates were isolated and screened for the loss of the plasmid-backbone-located antibiotic marker and by PCR for confirmation of the desired recombination event.

Mutant LAB cells carrying an FRT-flanked *erm* gene were transformed with the pNZFLPasRNA_repA plasmid for excision of the *erm* resistance gene. Upon appearance of transformants on MRS agar plates supplemented with Cm, single colonies were inoculated into MRS broth and incubated at 30 °C for 6 h for constitutive expression of FLP recombinase. Cell suspensions were then serially diluted and plated onto MRS agar plates containing Cm for propagation. Unmarked mutants that have lost the *erm* gene via FRT-FLP excision were identified by replica plating on MRS agar plates containing Cm or Em. Colonies that did not grow on MRS plates supplemented with Em were screened by PCR to confirm *erm* gene excision.

To generate unmarked mutants that would allow for further genetic manipulation using the nisin-MazF counter-selection marker system, cells carrying the FLP recombinase pNZFLPasRNA_repA plasmid were induced with nisin to stimulate loss of this plasmid. Cells carrying the pNZFLPasRNA_repA plasmid were inoculated into MRS broth supplemented with nisin, followed by incubation at 30 °C for 6 h for expression of the asRNA transcript. After incubation, the cell suspensions were serially diluted, and plated onto MRS agar plates not supplemented with Cm and incubated at 30 °C for 24 h. Mutant cells that have lost the pNZFLPasRNA_repA plasmid were identified by replica plating on MRS agar plates supplemented with and without Cm. Colonies that showed no growth on MRS plates supplemented with Cm indicated plasmid loss.

## Additional file

**Additional file 1: Text S1.** Construction of integration vectors; *mCherry* reporter gene expression in LAB using the nisin-inducible promoter; Detection of *in vitro* bioluminescence. **Table S1.** Bacterial strains and plasmids used in this study. **Table S2.** Oligonucleotides utilized in this study. **Fig. S1.** Optimization of nisin-controlled *mazF* gene expression in *L. plantarum* 423. **Fig. S2.** Plates showing the effect of PnisA promoter-controlled MazF protein and PnisA promoter-controlled mCherry fluorescence protein expression in LAB. **Fig. S3.** Schematic representing the construction of the pNZKOmunA::CatFfluc integrative plasmid. **Fig. S4.** Schematic representing the construction of the pNZKOplaA::ErmFfluc integrative plasmid. **Fig. S5.** Gene deletion and integration via homologous recombination into the genome of *L. plantarum* 423 at the *plaA* bacteriocin gene locus to create *L. plantarum* 423 *plaA*::*erm-ffluc*. **Fig. S6.** A workflow diagram showing the step-by-step design and protocol of the counterselection method developed in this study. **Fig. S7.** Zones of inhibition on plates overlaid with *L. monocytogenes* EGDe. **Fig. S8.** MRS agar plates showing bioluminescence emission of *ffluc* luciferase gene integration mutants. **Fig. S9.** Schematic representing the construction of the pNZKOsrtA::FRTerm integrative plasmid. **Fig. S10.** Schematic representing the construction of the pNZKOsrtC::FRTerm integrative plasmid. **Fig. S11.** Gene deletion and integration via homologous recombination into the genome of *E. mundtii* ST4SA at the *srtC* locus to create *E. mundtii* ST4SA *srtC*::FRT*erm*. **Fig. S12.** Schematic representing the construction of the MazF toxin expression plasmids pNZmazF and pNZmazFnisRK**. Fig. S13.** Schematic representing the construction of the pNZKIaap::FRTerm integrative plasmid. **Fig. S14.** Schematic representing the construction of plasmid pKSFRTErm containing the FRT-flanked *erm* gene. **Fig. S15.** Schematic representing the construction of plasmid pNZFLPasRNA_repA containing the *flp* recombinase gene and a *repA* asRNA fragment.

## Abbreviations

LAB: lactic acid bacteria
FLP: flippase
FRT: flippase recognition target
CRISPRs: clustered regularly interspaced palindromic repeats
Cas: CRISPR-associated
WT: wild-type
5-FU: 5-fluorouracil
PnisA: nisin inducible promoter
NICE: nisin-controlled expression system
mRNA: messenger RNA
Em: erythromycin
Cm: chloramphenicol
Amp: ampicillin
IVIS: in vivo imaging system
um: unmarked
asRNA: antisense RNA
gDNA: genomic DNA
KO: knockout
KI: knockin
AS: aggregation substance
ORF: open reading frame
ori: origin of replication
X-gal: 5-bromo-4-chloro-3-indolyl β-d-galactopyranoside
