# Supplementary material for: Development of a novel selection/counter-selection system for chromosomal gene integrations and deletions in lactic acid bacteria
Source: BMC Mol Biol. 2019 Mar 29;20:10. doi: 10.1186/s12867-019-0127-x (PMC6440147; doi:10.1186/s12867-019-0127-x)
Supplement: Supplementary file 1 — Additional file 1: Text S1. Construction of integration vectors; mCherry reporter gene expression in LAB using the nisin-inducible promoter; Detection of in vitro bioluminescence. Table S1. Bacterial strains and plasmids used in this study. Table S2. Oligonucleotides utilized in this study. Fig. S1. Optimization of nisin-controlled mazF gene expression in L. plantarum 423. Fig. S2. Plates showing the effect of PnisA promoter-controlled MazF protein and PnisA promoter-controlled mCherry fluorescence protein expression in LAB. Fig. S3. Schematic representing the construction of the pNZKOmunA::CatFfluc integrative plasmid. Fig. S4. Schematic representing the construction of the pNZKOplaA::ErmFfluc integrative plasmid. Fig. S5. Gene deletion and integration via homologous recombination into the genome of L. plantarum 423 at the plaA bacteriocin gene locus to create L. plantarum 423 plaA::erm-ffluc. Fig. S6. A workflow diagram showing the step-by-step design and protocol of the counterselection method developed in this study. Fig. S7. Zones of inhibition on plates overlaid with L. monocytogenes EGDe. Fig. S8. MRS agar plates showing bioluminescence emission of ffluc luciferase gene integration mutants. Fig. S9. Schematic representing the construction of the pNZKOsrtA::FRTerm integrative plasmid. Fig. S10. Schematic representing the construction of the pNZKOsrtC::FRTerm integrative plasmid. Fig. S11. Gene deletion and integration via homologous recombination into the genome of E. mundtii ST4SA at the srtC locus to create E. mundtii ST4SA srtC::FRTerm. Fig. S12. Schematic representing the construction of the MazF toxin expression plasmids pNZmazF and pNZmazFnisRK. Fig. S13. Schematic representing the construction of the pNZKIaap::FRTerm integrative plasmid. Fig. S14. Schematic representing the construction of plasmid pKSFRTErm containing the FRT-flanked erm gene. Fig. S15. Schematic representing the construction of plasmid pNZFLPasRNA_repA containing the flp recombinase ge [file 12867_2019_127_MOESM1_ESM.pdf]

Additional file 1

Development of a Novel Selection/Counter-selection System for Chromosomal Gene Integrations and Deletions in Lactic Acid Bacteria

Winschau F. Van Zyl, Leon M. T. Dicks[#], Shelly M. Deane

#Correspondence: Leon M. T. Dicks: lmtd@sun.ac.za

Additional file 1: Text S1

Construction of integration vectors

The *L. plantarum* 423 *plaA* bacteriocin gene knockout (KO) plasmid was constructed by PCR, amplifying the complete 2012 bp region of homology that included the *plaA* open reading frame (ORF), using primers plaAKO1 and plaAKO2 (Supplementary Figure S6). The generated amplicon was triple digested with EcoRI, HpaI and XbaI, resulting in the removal of 108 bp from the *plaA* ORF, ligated to a blunt-ended *erm-ffluc* gene cassette and pBluescriptKS digested with EcoRI and XbaI, yielding plasmid pKSplaA::ErmFfluc. The 3204 bp *erm-ffluc* gene cassette contained the *erm* gene for erythromycin resistance, the firefly luciferase gene (*ffluc*) from *Photinus pyralis* fused to the strong constitutive *L. plantarum ldh* gene (Pldh) promoter, generated via PCR using primer pair erm1/fluc2 and plasmid pNZErmFfluc423 (Supplementary Table S1) as source DNA. The pKSplaA::ErmFfluc construct was used as template for PCR amplification of a 5013 bp amplicon containing the *plaA* upstream, *plaA* downstream and *erm-ffluc* gene cassette using primer pair plaAKO1/plaAKO2. Next, the full- length blunt-ended 5013 bp PCR fragment

was cloned into the destination plasmid pNZmazFnisRK**,** before digestion of the plasmid with BglII and filling in the 5' sticky ends, yielding plasmid pNZKOplaA::ErmFfluc.

The pNZKOsrtC::FRTerm plasmid, designed for disruption of the *E. mundtii* ST4SA *sortase C* (*srtC*) gene was constructed as follows. Two regions of homology (~1 kb upstream and ~0.4 kb downstream) flanking the *srtC* gene were PCR amplified using primer pairs srtCKO1for/srtCKO1rev and srtCKO2for/srtCKO2rev, respectively (Supplementary Figure S7). The generated fragments were digested with HindIII/HpaI and HpaI/XbaI, ligated to the blunt-ended FRT-*erm* gene and cloned to the HindIII/XbaI double-digested pBluescriptKS plasmid, yielding plasmid pKSsrtC::FRTerm. Finally, the complete FRT-*erm* –*srtC* and flanking region was amplified from pKSsrtC::FRTerm using primer pair srtCKO1for/srtCKO2rev and cloned into the HindIII-linearized and blunt-ended plasmid pNZmazFnisRK, to yield plasmid pNZKOsrtC::FRTerm.

mCherry reporter gene expression in LAB using the nisin-inducible promoter

To demonstrate the utility of the nisin-induction system using a reporter gene, *Lc. lactis* pNZ9000 transformed with plasmid pNZCherry, *L. plantarum* 423 and *E. mundtii* ST4SA each transformed with plasmid pNZCherrynisRK were grown in MRS or M17 broth for 12 h (Supplementary Table S1). Each 12-h old culture was then serially diluted and plated onto MRS or M17 agar plates supplemented with or without nisin. Expression of the *mCherry* reporter gene was confirmed visually by a colony color change from white to pink or purple. Strains harboring the empty pNZ8048 vector were used as controls.

Detection of *in vitro* bioluminescence

Lactobacillus plantarum 423 and *E. mundtii* ST4SA recombinant strains carrying the *ffluc* bioluminescence gene were analyzed on agar plates for bioluminescence emission using the

Caliper *in vivo* imaging system (IVIS; Caliper Life Sciences, Hopkinton, MA, USA). Cultures were grown for 12 h in MRS broth, plated onto MRS agar plates using sterile swabs and incubated at 30°C for 24 h. Prior to imaging, one ml of beetle D-luciferin potassium salt substrate (Anatech Instruments, Bellville, South Africa) at 150 µg/ml dissolved in phosphate-buffered saline (PBS) was added directly to the colonies.

Additional file 1: Table S1: Bacterial strains and plasmids used in this study

Strain or plasmid	Description	Reference or source
Strains		
Lactobacillus plantarum		
423 pNZ8048	Contains the pNZ8048 plasmid; Cm^R	This study
423 pNZCherrynisRK	Contains the pNZCherrynisRK plasmid; Cm^R	This study
423 bac$^-$	Derivative of *L. plantarum* 423, cured of the plasmid (pPLA4) harboring the *plaA* bacteriocin gene	[44]
Enterococcus mundtii		
ST4SA pNZ8048	Contains the pNZ8048 plasmid; Cm^R	This study
ST4SA pNZCherrynisRK	Contains the pNZCherrynisRK plasmid; Cm^R	This study
Lactococcus lactis		
pNZ9000 pNZCherry	Contains the pNZCherry plasmid; Cm^R	This study
Listeria monocytogenes		
EGDe	Food-borne clinical pathogen; harbors the pPL2 lux plasmid; Cm^R	Caliper Life Sciences, Hopkinton, MA, U.S.A.
Plasmids		
pNZErmFluc423	pNZ8048 vector carrying the *erm* gene fused to the *Photinus pyralis* firefly luciferase gene (*ffluc*) under the control of the constitutive *L. plantarum* 423 lactate dehydrogenase (*ldh*) gene promoter (Pldh); Em^R, Cm^R	This study
pGKVCatFflucST4SA	pGKV223D vector carrying the *cat* gene fused to the *ffluc* gene under the control of the constitutive *E. mundtii* ST4SA lactate dehydrogenase (*ldh*) gene promoter (Pstldh); Em^R, Cm^R	This study
pKSplaA::ErmFfluc	pBluescriptKS plasmid carrying the *erm-ffluc* gene cassette flanked by *L. plantarum* 423 *plaA* gene regions of homology ; Em^R, Amp^R	This study
pKSmunA::CatFfluc	pBluescriptKS plasmid carrying the *cat-ffluc* gene cassette flanked by *E. mundtii* ST4SA *munA* gene regions of homology; Cm^R, Amp^R	This study
pKSaap::FRTerm	pBluescriptKS plasmid carrying the FRT-flanked *erm* gene flanked by *L. plantarum* 423 *aap* gene regions of homology ; Em^R, Amp^R	This study

pKSsrtA::FRTerm	pBluescriptKS plasmid carrying the FRT-flanked *erm* gene flanked by *E. mundtii* ST4SA *srtA* gene regions of homology ; Em^R, Amp^R	This study
pKSsrtC::FRTerm	pBluescriptKS plasmid carrying the FRT-flanked *erm* gene flanked by *E. mundtii* ST4SA *srtC* gene regions of homology; Em^R, Amp^R	This study
pNZCherry	pNZ8048 vector carrying the *mCherry* fluorescence gene under the control of the PnisA promoter; Cm^R	This study
pNZCherrynisRK	pNZnisRK vector carrying *mCherry* fluorescence gene under the control of the PnisA promoter; Cm^R	This study

Cm^R, chloramphenicol resistance; Em^R, erythromycin resistance; Amp^R, ampicillin resistance

References

Van Reenen, C.A., Van Zyl, W.H. & Dicks, L.M.T. (2006) Expression of the immunity protein of plantaricin 423, produced by *Lactobacillus plantarum* 423, and analysis of the plasmid encoding the bacteriocin. *Appl. Environ. Microbiol*. 72, 7644–7651.

Additional file 1: Table S2: Oligonucleotides utilized in this study

Target	Primer	Sequence (5' to 3')[†]	Restriction sites	Product size (bp**)**
nisR-nisK	nisRK1	GCGC**AAGCTT**CCCCGGCTTTAGGTATAG	HindIII	2162
	nisRK2	ATCC**CTCGAG**TTACTTTTTTATTTTTAGGATA	XhoI	
mazF	mazF1	GCTTGGAT**CCATGG**TAAGCCGATACGTACC	NcoI	342
	mazF2	AGTC**AAGCTT**CTACCCAATCAGTACGTTAAT T	HindIII	
erm-ffluc gene cassette	erm1	ATAC**GAATTC**CACGACCAAAACTATAAAACC	EcoRI	3204
	fluc2	GCGC**AAGCTT**CACAATTTCGACTTGCCA	HindIII	
plaA KO region	plaAKO1	**GAATTC**GTGATATATAATGATGGAATTTATTC	EcoRI	2012
	plaAKO2	**TCTAGA**GCCTTAGCGTACTTATCCCAGGC	XbaI	
munA KO region	munAKO1	GCCG**GAATTC**GTTTATACGATTAGTGGAATT A	EcoRI	1809
	munAKO2	ATAC**TCTAGA**GACAATATCTCCAGTTTT	XbaI	
cat-ffluc gene cassette	cat1	G**GAATTC**GGATCCATTCTAATGAAG	EcoRI	2349
	fluc2	GCGC**AAGCTT**CACAATTTCGACTTGCCA	HindIII	
aap KI region	aapKO1	GCCG**GAATTC**GAGTACTGTGACGACAGCTA	EcoRI	1848
	aapKO2	ATAC**TCTAGA**TAATCGTCCGGGTAACCGTT	XbaI	
FRT-*erm* gene	M13for	GTAAAACGACGGCCAG	blunt	1458
	M13rev	CAGGAAACAGCTATGA	blunt	
srtA upstream region	srtAKO1for	CACC**AAGCTT**GTAGGTTACTTCATCAACGG	HindIII	1011
	srtAKO1rev	GATA**GTTAAC**TAGTAGAAACAATAGGAAAT	HpaI	
srtA down-stream region	srtAKO2for	TATA**GTTAAC**AAAGAAGCCACCCAAGAAAT	HpaI	376
	srtAKO2rev	GATA**TCTAGA**GCCGAAAAATTTACTGATGA	XbaI	
srtC upstream region	srtCKO1for	GAGG**AAGCTT**ATTAGATGGGCAAGACGTAG	HindIII	1011
	srtCKO1rev	GATA**GTTAAC**CCGATAAAAAATACCGAGAA	HpaI	
srtC down-stream region	srtCKO2for	CACC**GTTAAC**TTTTTGTTGCTATTTCTTAT	HpaI	1035
	srtCKO2rev	CACC**TCTAGA**AGATTATAATTTGAATTTTA	XbaI	

FRT target 1	FRTfor1	AGAT**<u>GGATCC</u>**CTCGTTTTCGGAAACGCTTT	BamHI	121
	FRTrev1	GATA**<u>CTGCAG</u>**TTCAGAGCGCTTTTGGTTTT	PstI	
FRT target 2	FRTfor2	AGAT**<u>AAGCTT</u>**CTCGTTTTCGGAAACGCTTT	HindIII	121
	FRTrev2	GATA**<u>GTCGAC</u>**TTCAGAGCGCTTTTGGTTTT	SalI	
erm gene	erm1	ATAC**<u>GAATTC</u>**CACGACCAAAACTATAAAACC	EcoRI	1063
	erm 2	GCTT**<u>GAATTC</u>**TTACTTATTAAATAATTTATAG		
FLP gene	FLPfor	GATA**<u>CCATGG</u>**ATGCCACAATTTGGTATATT	NcoI	1293
	FLPrev	AGTA**<u>GTCGAC</u>**TTATATGCGTCTATTTATGT	SalI	
asRNA repA	asRNAfor	ATAT**<u>CCATGG</u>**TTCATATGAACCTTTGAT	NcoI	350
	asRNArev	CACG**<u>AAGCTT</u>**GATAAGGTAATTATATCAT		
Pldh-FLP amplicon	Pldh1	**<u>GAATTC</u>**AATCTTCTCACCGTCT	EcoRI	1794
	FLPrev	AGTA**<u>GTCGAC</u>**TTATATGCGTCTATTTATGT	SalI	
plaA KO integration region	plaAKOc	AATATCTTCGTTGCTGTGAT	-	5118
	plaAKO2	**<u>TCTAGA</u>**GCCTTAGCGTACTTATCCCAGGC	XbaI	
munA KO intergration region	munAKOc	ATTCTTGAGAACATTCCACA	-	4190
	munAKO2	ATAC**<u>TCTAGA</u>**GACAATATCTCCAGTTTT	XbaI	
aap KI integration region	aapKOc	GGCGCCAGCAGCCAACTCAA	-	3696
	aapKOc2	CGTAGCCCTCATGGCTCAGA	-	
aap KI unmarked integration region	aapKOc	GGCGCCAGCAGCCAACTCAA	-	2573
	aapKOc2	CGTAGCCCTCATGGCTCAGA	-	
s*rtA* KO integ ration region	srtAKOc	GATGGTTTTGTTTATTCGAA	-	3853
	srtAKO2rev	GATATCTAGAGCCGAAAAATTTACTGATGA	-	
srtC KO integration region	srtCKOc	GAAGGAACGCTGAAGGTCAA	-	3183
	srtCKOc2	AAACTAGTCCTGTCGTTCCTT	-	

†: Bold and underlined sequences indicate restriction sites; Full target gene names are listed in main text and Table 2; KO: knockout; KOc: knockout confirmation; for: forward; rev: reverse.

(a)

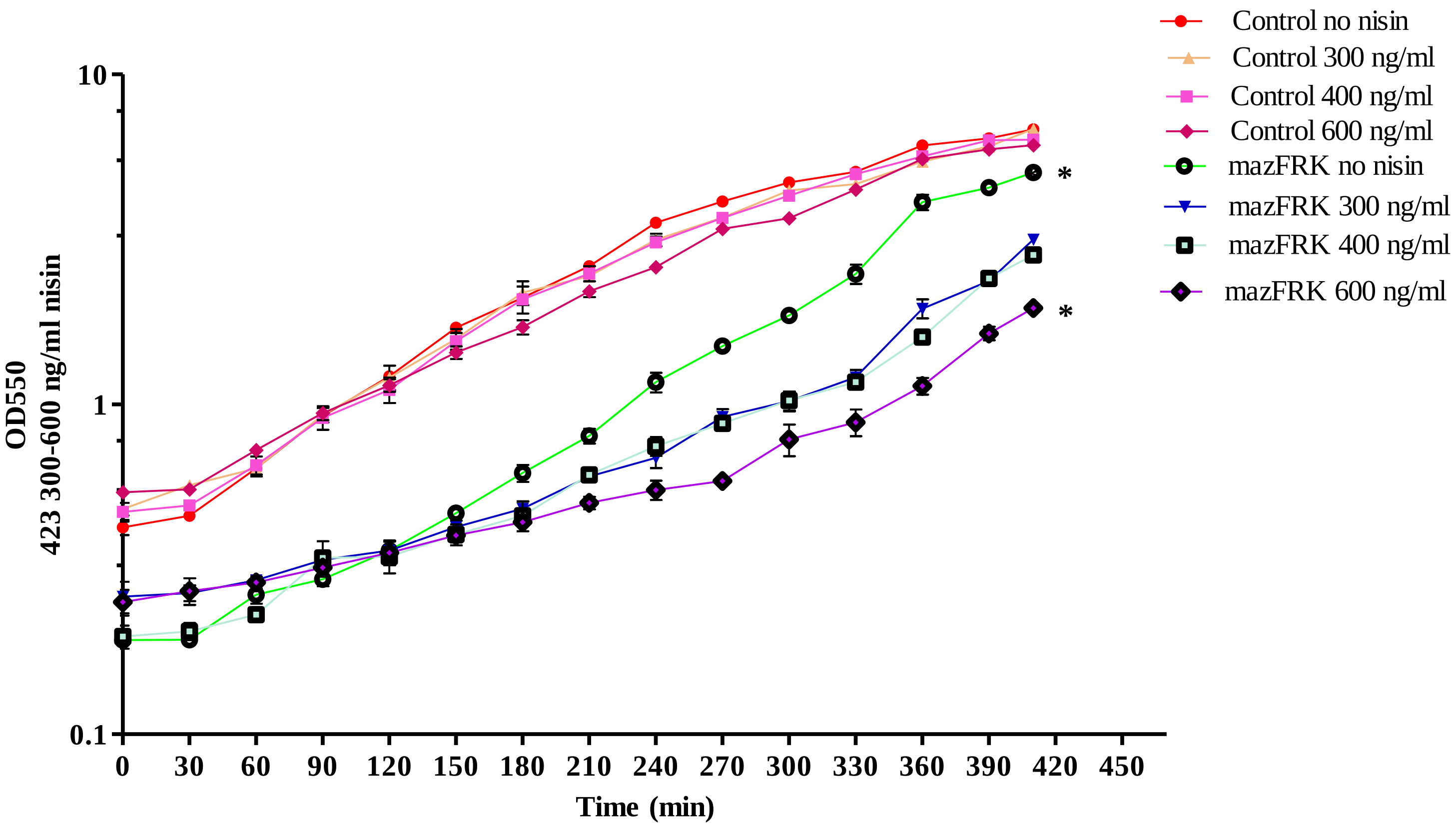

(b)

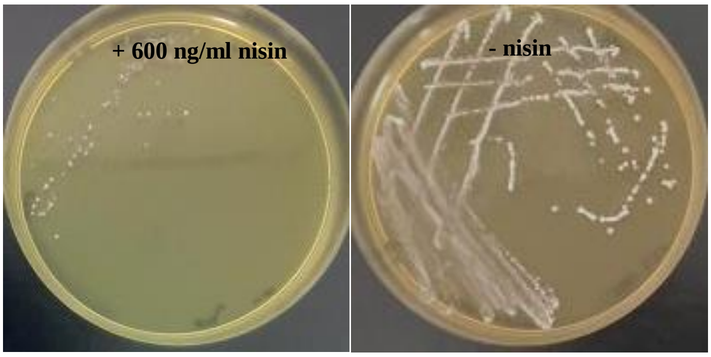

Additional file 1: Fig. S1. Optimization of nisin-controlled *maz*F gene expression in *L. plantarum* 423. **(a)** Growth comparison of *L. plantarum* 423 transformed with the empty pNZ8048 vector (control) and *L. plantarum* 423 transformed with the PnisA controlled *maz*F gene pNZmazFnisRK plasmid in sub-inhibitory concentrations of nisin (0 - 600 ng/ml). Significant differences (P<0.05; Kruskal-Wallis nonparametric test) between mazFRK no nisin and mazFRK 600 ng/ml are

indicated with an asterisk. **(b)** MRS agar plates representative of the effect of MazF protein expression in *L. plantarum* 423 harboring the pNZmazFnisRK plasmid in the absence of nisin (-nisin) and in the presence of nisin (+600 ng/ml nisin).

(a)

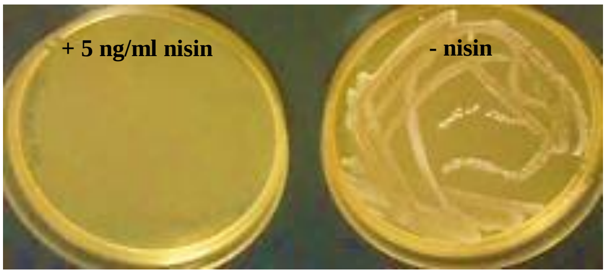

(b)

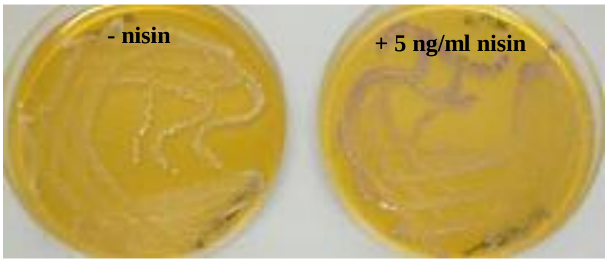

©

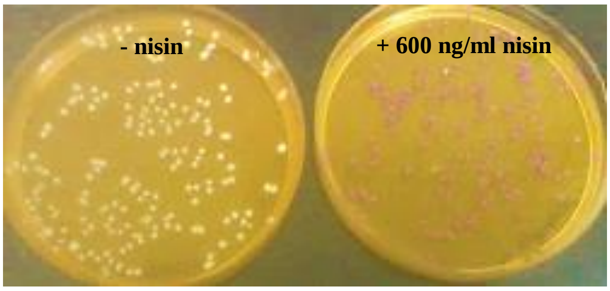

(d0

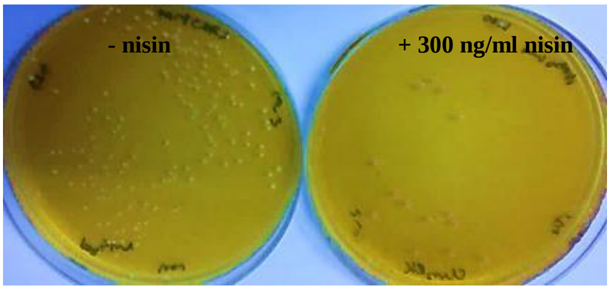

Additional file 1: Fig. S2. Plates showing the effect of PnisA promoter-controlled MazF protein expression in *Lc. lactis* pNZ9000 and PnisA promoter-controlled mCherry fluorescence protein expression in *Lc. lactis* pNZ9000, *L. plantarum* 423 and *E. mundtii* ST4SA. **(a)** M17 agar plates representative of the effect of MazF protein expression in *Lc. lactis* pNZ9000 harboring the pNZmazF plasmid in the absence of nisin (-nisin) and in the presence of nisin (+5 ng/ml nisin). **(b)** M17 agar plates showing the expression of the *mCherry* fluorescence gene in *Lc. lactis* pNZ9000 harboring the pNZCherry plasmid in the absence of nisin (-nisin) and in the presence of nisin (+5 ng/ml nisin). **(c)** MRS agar plates showing the expression of the *mCherry* fluorescence

gene in *L. plantarum* 423 harboring the pNZCherrynisRK plasmid in the absence of nisin (-nisin) and in the presence of nisin (+600 ng/ml nisin). **(d)** MRS agar plates showing the expression of the *mCherry* fluorescence gene in *E. mundtii* ST4SA harboring the pNZCherrynisRK plasmid in the absence of nisin (-nisin) and in the presence of nisin (+300 ng/ml nisin).

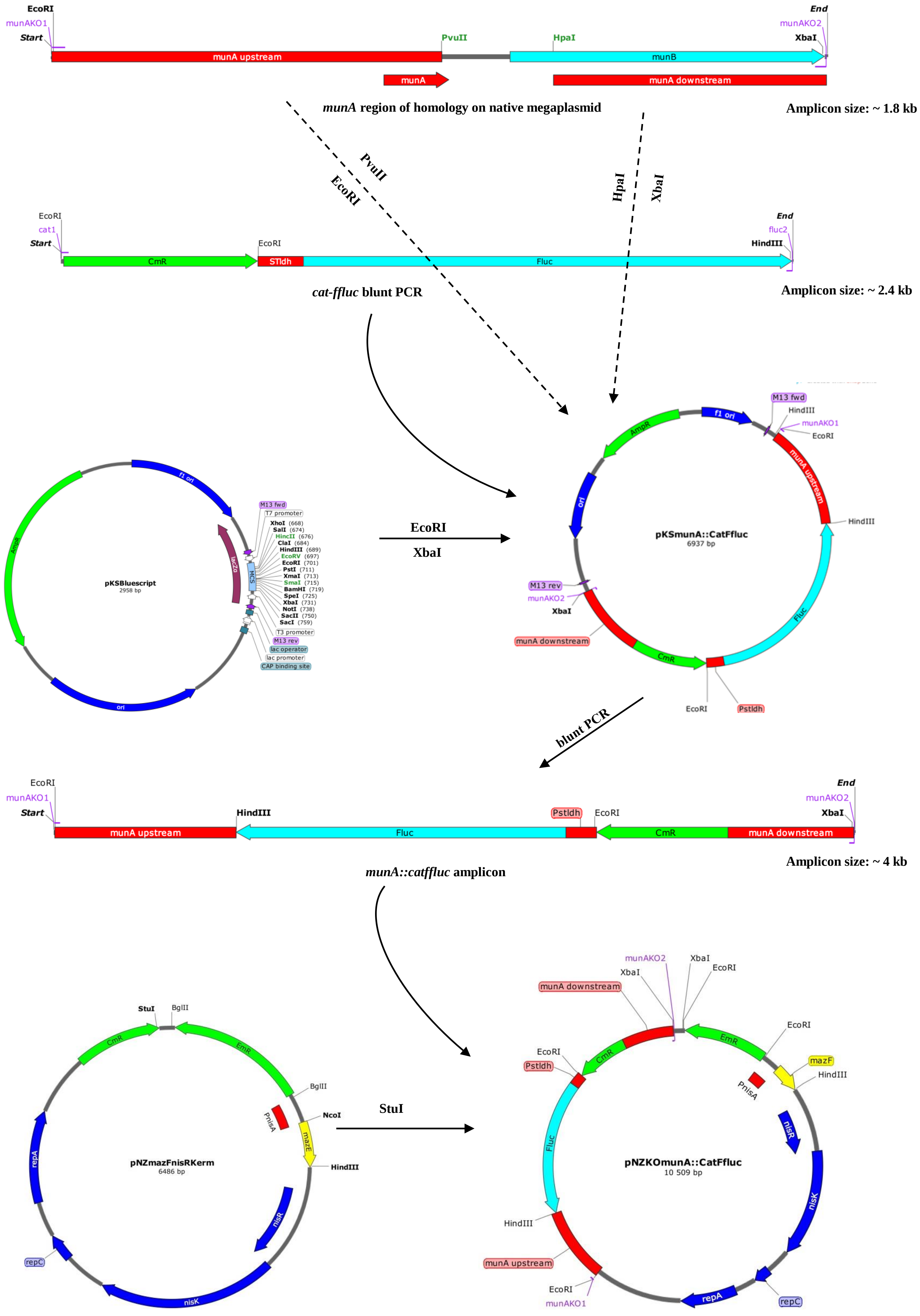
EcoRI
munAKO1
Start
munA upstream
PvuII
HpaI
munB
End
munAKO2
XbaI
munA
munA downstream
munA region of homology on native megaplasmid
Amplicon size: ~ 1.8 kb
PvuII
EcoRI
HpaI
XbaI
EcoRI
cat1
Start
CmR
STldh
Fluc
End
fluc2
HindIII
cat-ffluc blunt PCR
Amplicon size: ~ 2.4 kb
pKSBluescript
2958 bp
f1 ori
AmpR
lacZα
MCS
ori
M13 fwd
T7 promoter
XhoI (668)
SalI (674)
HincII (676)
ClaI (684)
HindIII (689)
EcoRV (697)
EcoRI (701)
PstI (711)
XmaI (713)
SmaI (715)
BamHI (719)
SpeI (725)
XbaI (731)
NotI (738)
SacII (750)
SacI (759)
T3 promoter
M13 rev
lac operator
lac promoter
CAP binding site
EcoRI
XbaI
pKSmunA::CatFfluc
6937 bp
M13 fwd
HindIII
munAKO1
EcoRI
f1 ori
AmpR
ori
munA upstream
HindIII
M13 rev
munAKO2
XbaI
munA downstream
CmR
Fluc
EcoRI
Pstldh
blunt PCR
EcoRI
munAKO1
Start
munA upstream
HindIII
Fluc
Pstldh
EcoRI
CmR
munA downstream
End
munAKO2
XbaI
munA::catffluc amplicon
Amplicon size: ~ 4 kb
pNZmazFnisRKerm
6486 bp
StuI
BglII
CmR
EmR
BglII
PnisA
NcoI
mazE
HindIII
repA
repC
nisR
nisK
StuI
pNZKOmunA::CatFfluc
10 509 bp
munAKO2
XbaI
XbaI
EcoRI
munA downstream
EcoRI
Pstldh
CmR
EmR
EcoRI
mazF
HindIII
PnisA
Fluc
nisR
nisK
HindIII
munA upstream
EcoRI
munAKO1
repA
repC

Additional file 1: Fig. S3. Schematic representing the construction of the pNZKOmunA::CatFfluc integrative plasmid containing the *cat* gene and two flanking sequences of the *munA* bacteriocin gene from *E. mundtii* ST4SA. Relevant genes, restriction sites and PCR primers are shown. Construction of the plasmid is best understood by referring to “Materials and Methods” in the main text.

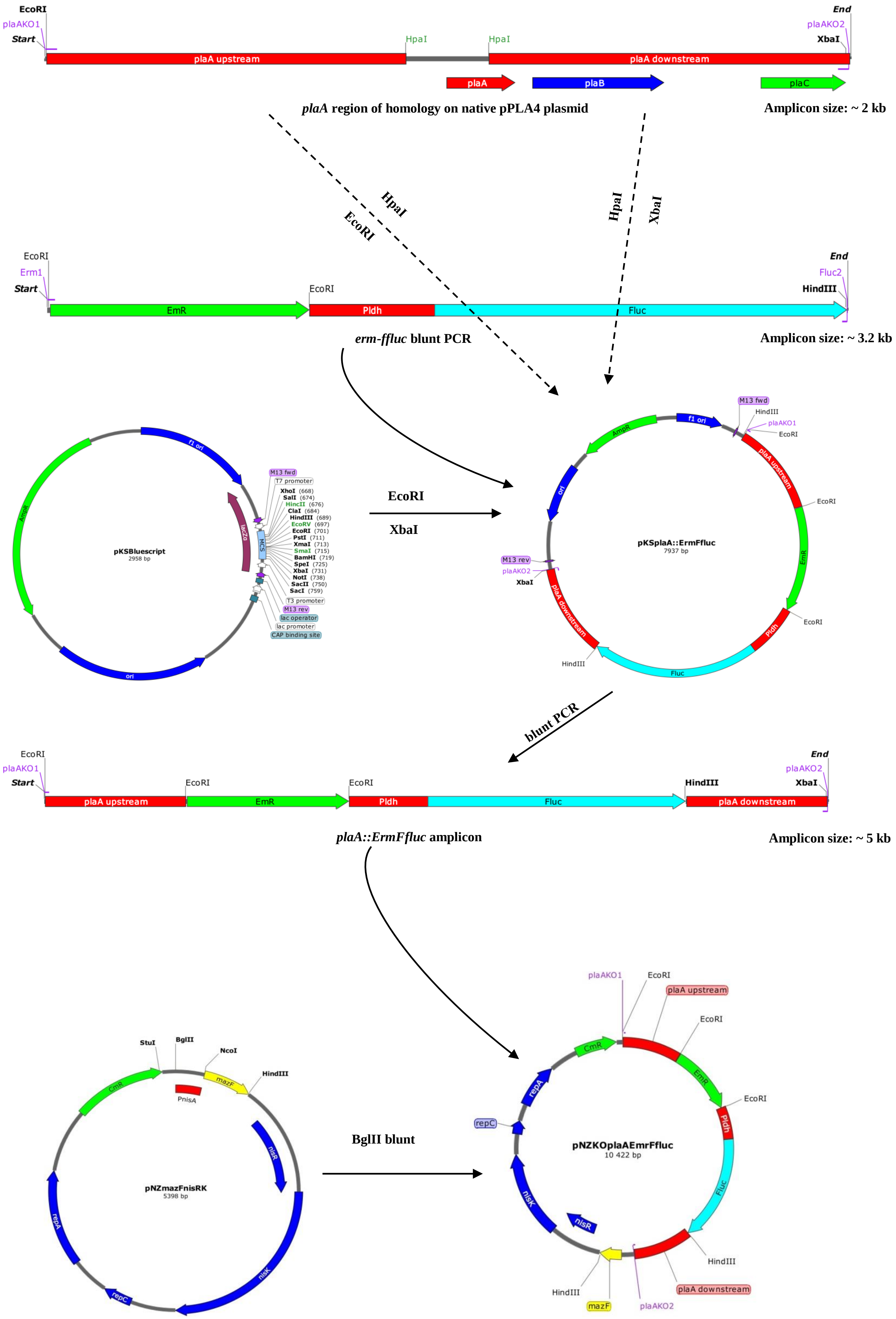

EcoRI
plaAKO1
Start
HpaI
HpaI
End
plaAKO2
XbaI
plaA upstream
plaA downstream
plaA
plaB
plaC
plaA region of homology on native pPLA4 plasmid
Amplicon size: ~ 2 kb
HpaI
EcoRI
HpaI
XbaI
EcoRI
Erm1
Start
EcoRI
End
Fluc2
HindIII
EmR
Pldh
Fluc
erm-ffluc blunt PCR
Amplicon size: ~ 3.2 kb
pKSBluescript
2958 bp
f1 ori
AmpR
lacZα
MCS
ori
M13 fwd
T7 promoter
XhoI (668)
SalI (674)
HincII (676)
ClaI (684)
HindIII (689)
EcoRV (697)
EcoRI (701)
PstI (711)
XmaI (713)
SmaI (715)
BamHI (719)
SpeI (725)
XbaI (731)
NotI (738)
SacII (750)
SacI (759)
T3 promoter
M13 rev
lac operator
lac promoter
CAP binding site
EcoRI
XbaI
pKSplaA::ErmFfluc
7937 bp
M13 fwd
HindIII
plaAKO1
EcoRI
f1 ori
AmpR
ori
plaA upstream
EcoRI
EmR
EcoRI
Pldh
Fluc
HindIII
plaA downstream
M13 rev
plaAKO2
XbaI
blunt PCR
EcoRI
plaAKO1
Start
EcoRI
EcoRI
HindIII
End
plaAKO2
XbaI
plaA upstream
EmR
Pldh
Fluc
plaA downstream
plaA::ErmFfluc amplicon
Amplicon size: ~ 5 kb
pNZmazFnisRK
5398 bp
StuI
BglII
NcoI
HindIII
CmR
mazF
PnisA
nisR
nisK
repA
repC
BglII blunt
pNZKOplaAEmrFfluc
10 422 bp
plaAKO1
EcoRI
plaA upstream
EcoRI
EcoRI
CmR
EmR
Pldh
Fluc
repA
repC
nisK
nisR
HindIII
plaA downstream
HindIII
mazF
plaAKO2

Additional file 1: Fig. S4. Schematic representing the construction of the pNZKOplaA::ErmFfluc integrative plasmid containing the *erm* gene and two flanking sequences of the *plaA* bacteriocin gene from *L. plantarum* 423. Relevant genes, restriction sites and PCR primers are shown. Construction of the plasmid is best understood by referring to the Additional file 1 Text S1.

(a)

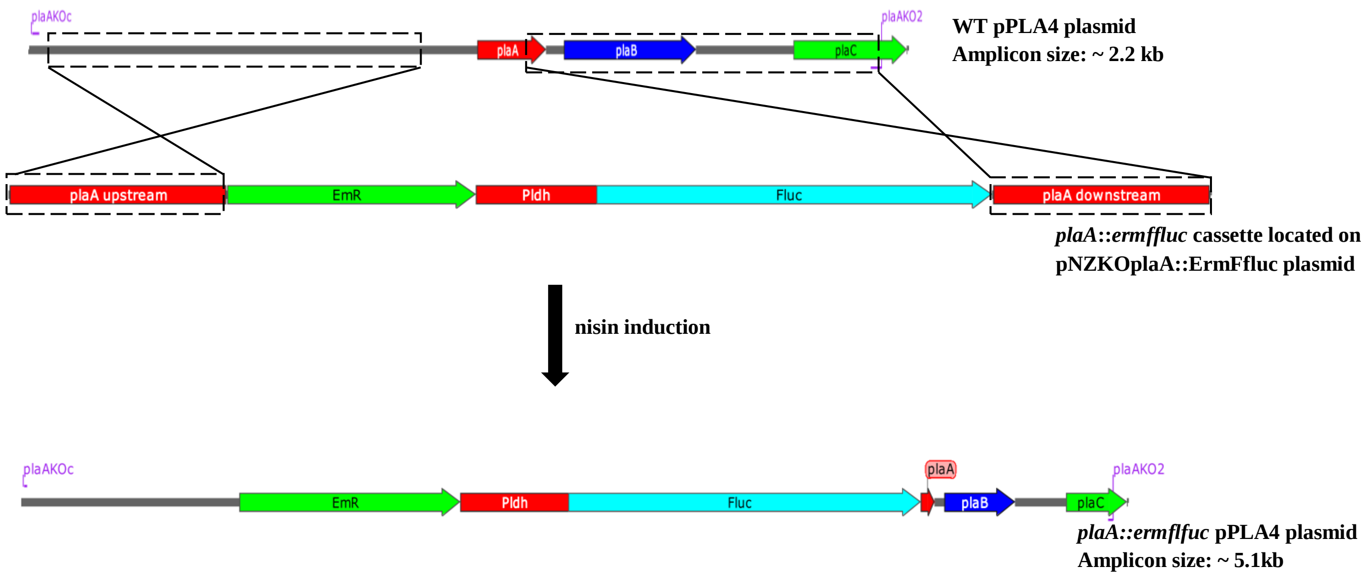

(b)

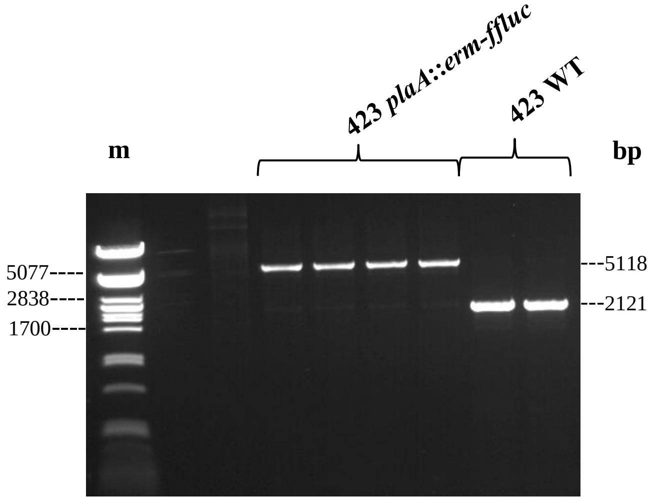

Additional file 1: Fig. S5. Gene deletion and integration via homologous recombination into the genome of *L. plantarum* 423 at the *plaA* bacteriocin gene locus to create *L. plantarum* 423 *plaA::erm-ffluc*. **(a)** Homologous recombination between the wild-type (WT) pPLA4 plasmid and the *plaA::ermffluc* cassette. Boxed regions show the upstream and downstream regions of homology (~ 0.9 kb) on plasmid pPLA4 and the pNZKOplaAErmFfluc KO vector. Cells harboring the *plaA* KO vector were selected on Cm and Em, followed by nisin induction for MazF toxin expression to select for mutants that have lost the plasmid backbone bearing *cat* and *mazF* genes. Double crossover mutants were selected and screened by PCR using the primer combinations indicated in purple. **(b)** PCR amplification of WT and *plaA* deletion and insertion mutants using the primer pair indicated in panel A. **(m)** Lambda DNA digested with PstI (NEB). Amplicons from four *plaA* mutant and two WT colonies, respectively, are shown.

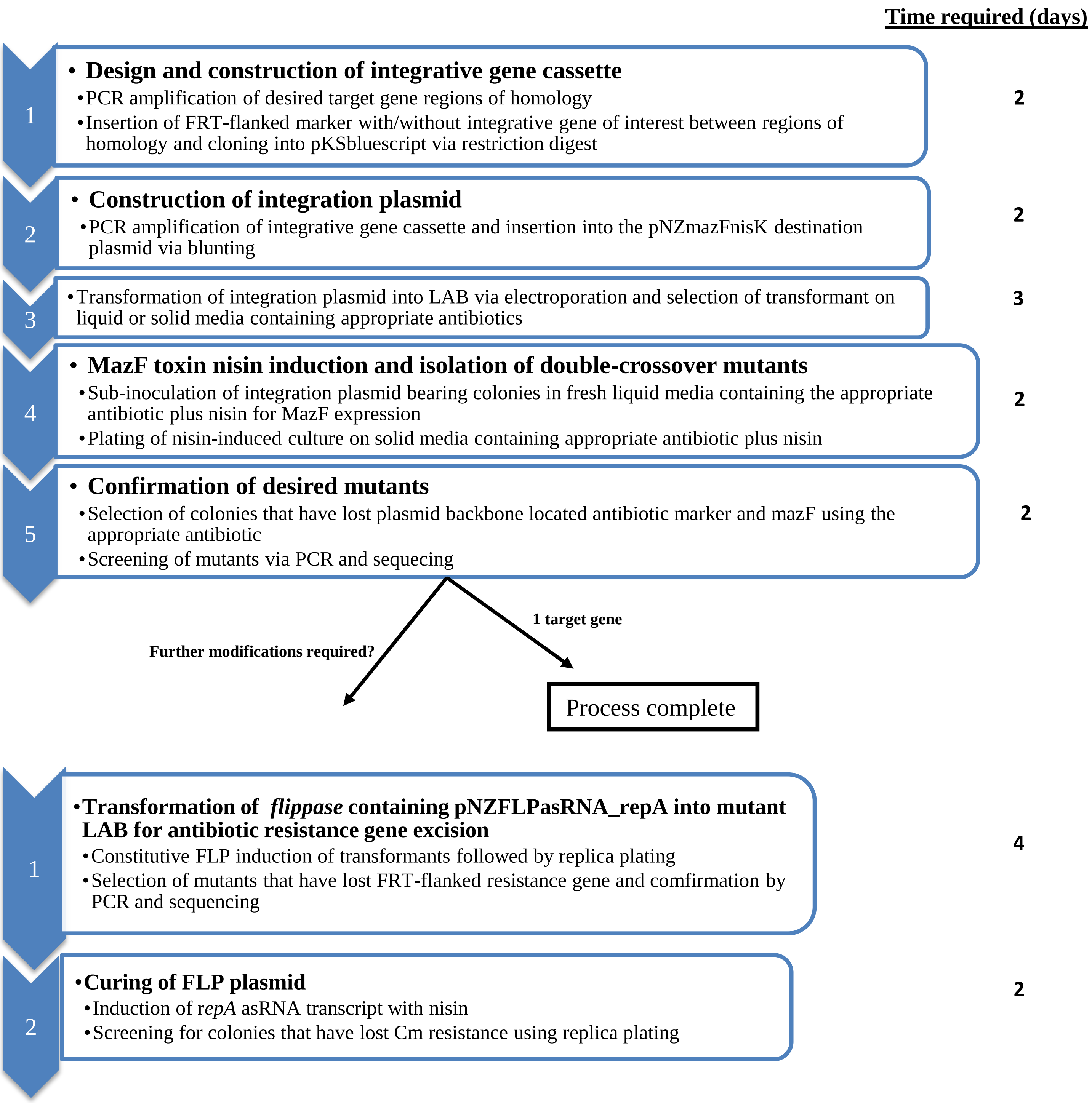

Additional file 1: Fig. S6. A workflow diagram showing the step-by-step design and protocol of the newly developed counterselection method for the deletion and inactivation of LAB genes or to introduce genes of interest followed by resistance marker recycling.

(a) (b)

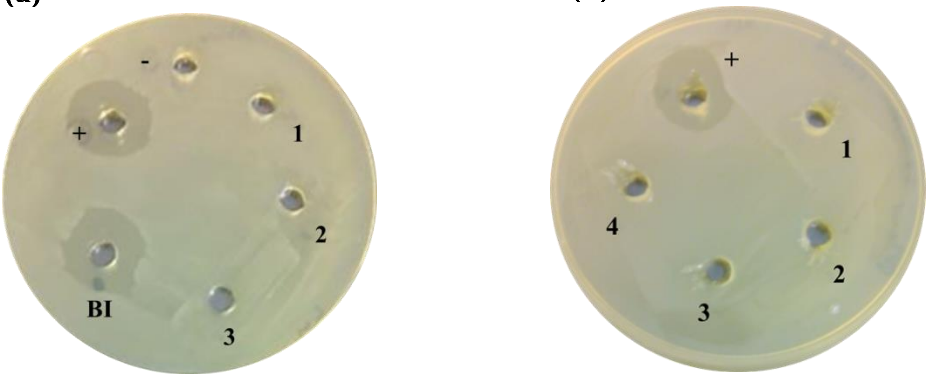

Additional file 1: Fig. S7. Zones of inhibition on plates overlaid with *L. monocytogenes* EGDe. **a.** (BI) Supernatant containing bacteriocin plantaricin 423 isolated from *L. plantarum* 423 transformed with KO plasmid pNZKOplaA::ErmFfluc before induction with nisin, (+) supernatant containing bacteriocin plantaricin 423 isolated from wild-type (WT) *L. plantarum* 423 as positive control, (-) supernatant lacking plantaricin 423 bacteriocin isolated from *L. plantarum* 423 bac- as negative control and supernatants (1, 2 & 3) lacking bacteriocin plantaricin 423 isolated from three *L. plantarum* 423 *plaA::erm-ffluc plaA* gene deletion mutants. **b.** (+) Supernatant containing bacteriocin mundticin ST4SA isolated from WT *E. mundtii* ST4SA as positive control and supernatants (1, 2, 3 & 4) lacking bacteriocin mundticin ST4SA isolated from four *E. mundtii* ST4SA *munA::cat-ffluc munA* gene deletion mutants.

(a)

(b)

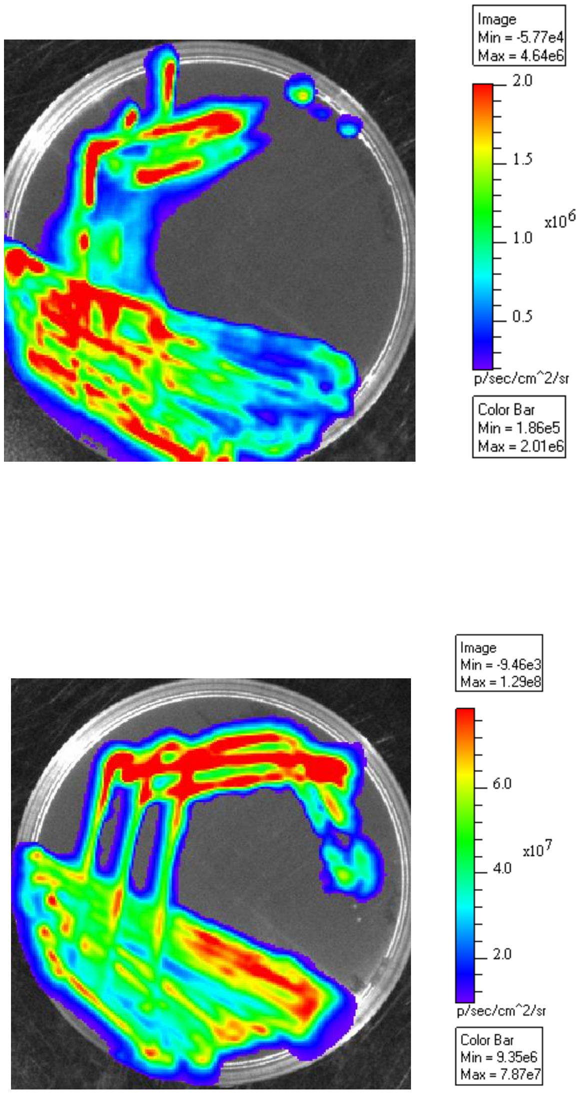

Additional file 1: Fig. S8. MRS agar plates showing bioluminescence emission of *ffluc* luciferase gene integration mutants. **(a)** Bioluminescent *L. plantarum* 423 *plaA::erm-ffluc* double-crossover mutant colonies harboring the *ffluc* gene integrated at the *plaA* locus. (b) Bioluminescent *E. mundtii* ST4SA *munA::cat-ffluc* double-crossover mutant colonies harboring the *ffluc* gene integrated at the *munA* locus. Images were generated using the Living Image® software program and representative scale bars (in photons per second) are indicated for each image (red, most intense and purple being the least intense).

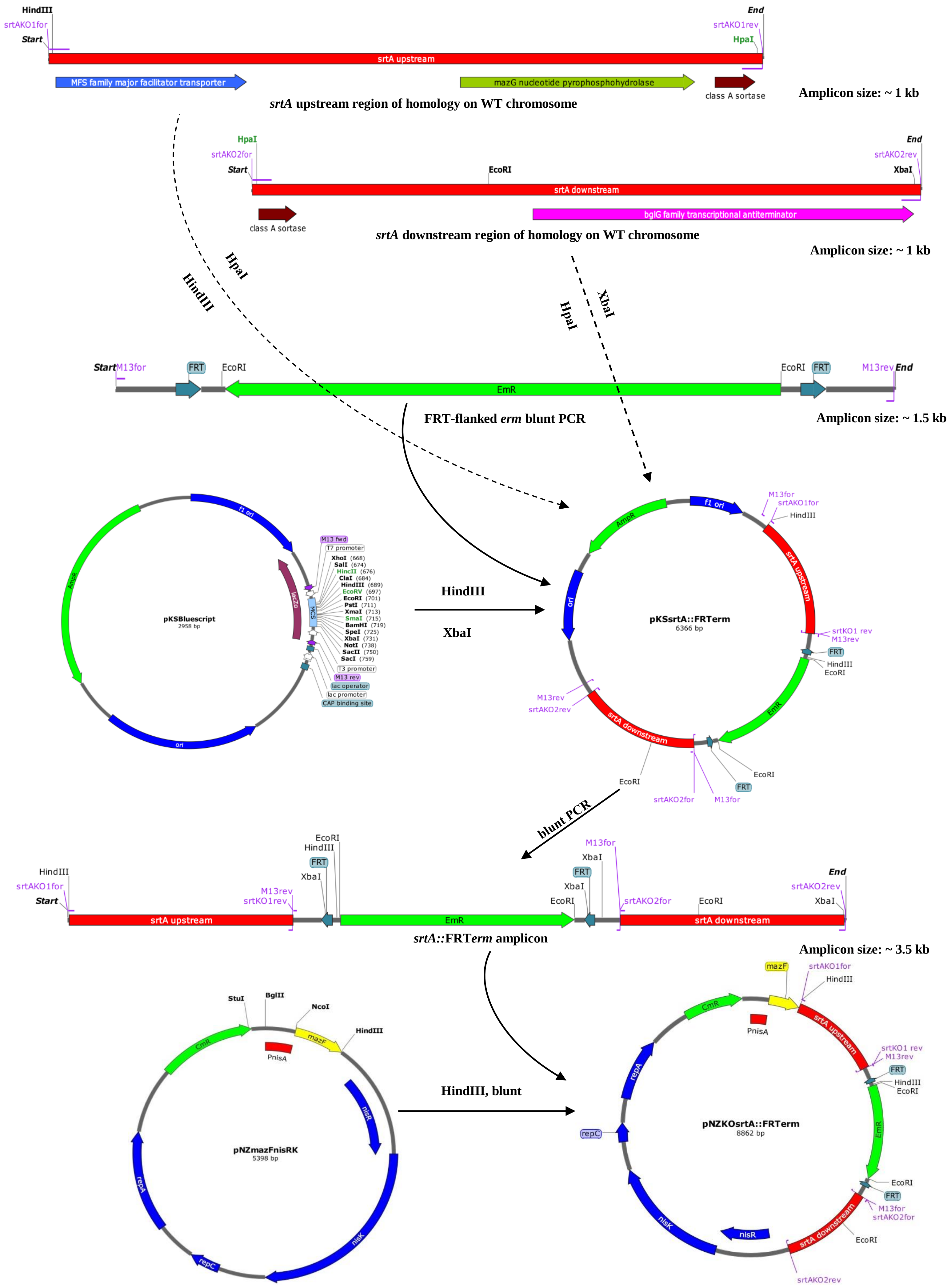
HindIII
srtAKO1for
Start
End
srtAKO1rev
HpaI
srtA upstream
MFS family major facilitator transporter
mazG nucleotide pyrophosphohydrolase
class A sortase
srtA upstream region of homology on WT chromosome
Amplicon size: ~ 1 kb
HpaI
srtAKO2for
Start
EcoRI
End
srtAKO2rev
XbaI
srtA downstream
class A sortase
bglG family transcriptional antiterminator
srtA downstream region of homology on WT chromosome
Amplicon size: ~ 1 kb
HpaI
HindIII
XbaI
HpaI
Start
M13for
FRT
EcoRI
EmR
EcoRI
FRT
M13rev
End
FRT-flanked erm blunt PCR
Amplicon size: ~ 1.5 kb
f1 ori
AmpR
lacZα
MCS
pKSBluescript
2958 bp
ori
M13 fwd
T7 promoter
XhoI (668)
SalI (674)
HincII (676)
ClaI (684)
HindIII (689)
EcoRV (697)
EcoRI (701)
PstI (711)
XmaI (713)
SmaI (715)
BamHI (719)
SpeI (725)
XbaI (731)
NotI (738)
SacII (750)
SacI (759)
T3 promoter
M13 rev
lac operator
lac promoter
CAP binding site
HindIII
XbaI
M13for
srtAKO1for
HindIII
AmpR
f1 ori
srtA upstream
ori
pKSsrtA::FRTerm
6366 bp
srtKO1 rev
M13rev
FRT
HindIII
EcoRI
EmR
M13rev
srtAKO2rev
srtA downstream
EcoRI
EcoRI
FRT
srtAKO2for
M13for
blunt PCR
EcoRI
HindIII
FRT
XbaI
HindIII
srtAKO1for
Start
M13rev
srtKO1rev
M13for
XbaI
FRT
XbaI
EcoRI
srtAKO2for
EcoRI
End
srtAKO2rev
XbaI
srtA upstream
EmR
srtA downstream
srtA::FRTerm amplicon
Amplicon size: ~ 3.5 kb
StuI
BglII
NcoI
HindIII
CmR
mazF
PnisA
nisR
pNZmazFnisRK
5398 bp
repA
nisK
repC
HindIII, blunt
mazF
srtAKO1for
HindIII
CmR
PnisA
srtA upstream
srtKO1 rev
M13rev
FRT
HindIII
EcoRI
repA
repC
pNZKOsrtA::FRTerm
8862 bp
EmR
EcoRI
FRT
M13for
srtAKO2for
nisK
nisR
srtA downstream
EcoRI
srtAKO2rev

Additional file 1: Fig. S9. Schematic representing the construction of the pNZKOsrtA::FRTerm integrative plasmid containing the *erm* gene and two flanking sequences of the *srtA* cell wall adhesion associated gene from *E. mundtii* ST4SA. Relevant genes, restriction sites and PCR primers are shown. Construction of the plasmid is best understood by referring to “Materials and Methods” in the main text.

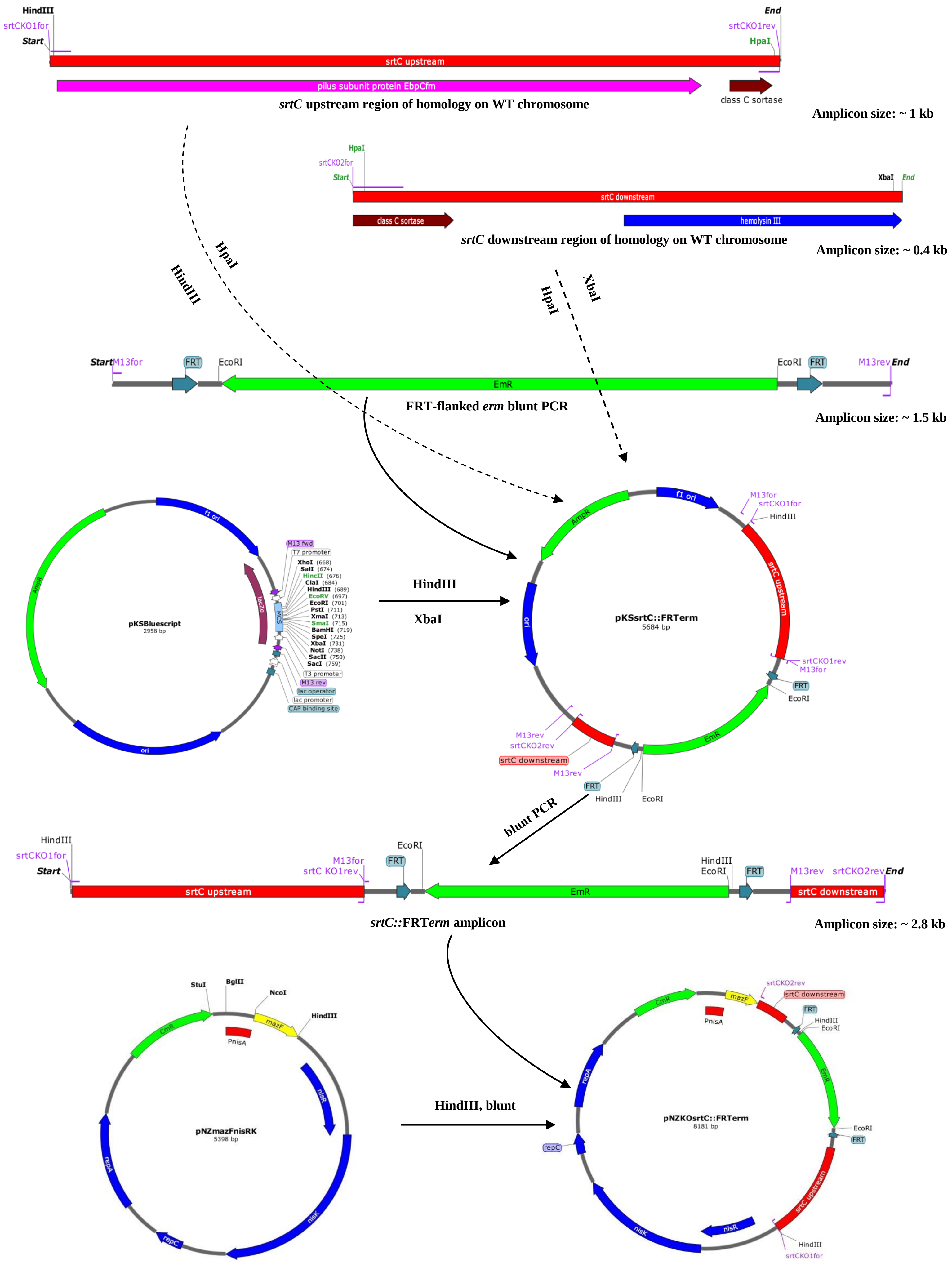

HindIII
srtCKO1for
Start
End
srtCKO1rev
HpaI
srtC upstream
pilus subunit protein EbpCfm
class C sortase
srtC upstream region of homology on WT chromosome
Amplicon size: ~ 1 kb
HpaI
srtCKO2for
Start
XbaI
End
srtC downstream
class C sortase
hemolysin III
srtC downstream region of homology on WT chromosome
Amplicon size: ~ 0.4 kb
HpaI
HindIII
XbaI
HpaI
Start
M13for
FRT
EcoRI
EmR
EcoRI
FRT
M13rev
End
FRT-flanked erm blunt PCR
Amplicon size: ~ 1.5 kb
pKSBluescript
2958 bp
f1 ori
AmpR
ori
lacZα
MCS
M13 fwd
T7 promoter
XhoI (668)
SalI (674)
HincII (676)
ClaI (684)
HindIII (689)
EcoRV (697)
EcoRI (701)
PstI (711)
XmaI (713)
SmaI (715)
BamHI (719)
SpeI (725)
XbaI (731)
NotI (738)
SacII (750)
SacI (759)
T3 promoter
M13 rev
lac operator
lac promoter
CAP binding site
HindIII
XbaI
pKSsrtC::FRTerm
5684 bp
f1 ori
AmpR
ori
M13for
srtCKO1for
HindIII
srtC upstream
srtCKO1rev
M13for
FRT
EcoRI
EmR
M13rev
srtCKO2rev
srtC downstream
M13rev
FRT
HindIII
EcoRI
blunt PCR
HindIII
srtCKO1for
Start
srtC upstream
M13for
srtC KO1rev
FRT
EcoRI
EmR
HindIII
EcoRI
FRT
M13rev
srtCKO2rev
End
srtC downstream
srtC::FRTerm amplicon
Amplicon size: ~ 2.8 kb
StuI
BglII
NcoI
HindIII
CmR
mazF
PnisA
nisR
nisK
repA
repC
pNZmazFnisRK
5398 bp
HindIII, blunt
pNZKOsrtC::FRTerm
8181 bp
CmR
mazF
PnisA
srtCKO2rev
srtC downstream
FRT
HindIII
EcoRI
EmR
EcoRI
FRT
srtC upstream
HindIII
srtCKO1for
nisR
nisK
repA
repC

Additional file 1: Fig. S10. Schematic representing the construction of the pNZKOsrtC::FRTerm integrative plasmid containing the *erm* gene and two flanking sequences of the *srtC* cell pilus associated gene from *E. mundtii* ST4SA. Relevant genes, restriction sites and PCR primers are shown. Construction of the plasmid is best understood by referring to the Additional file 1 Text S1.

(a)

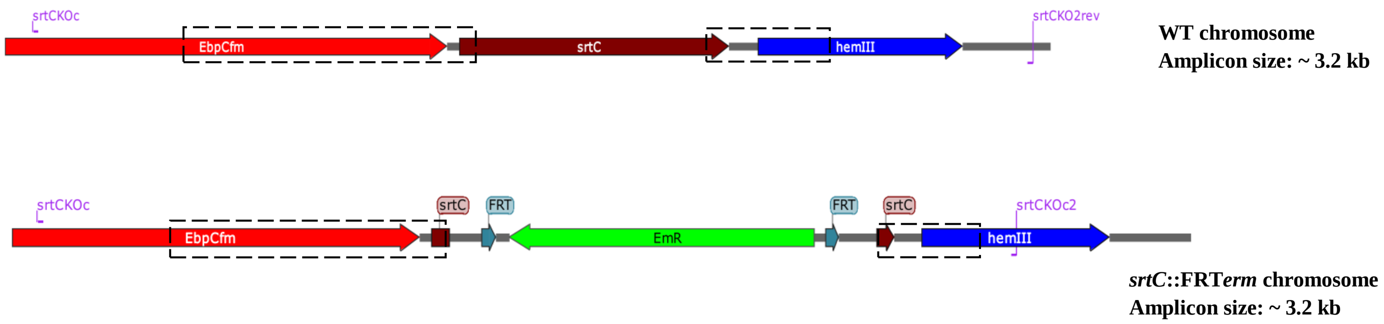

(b)

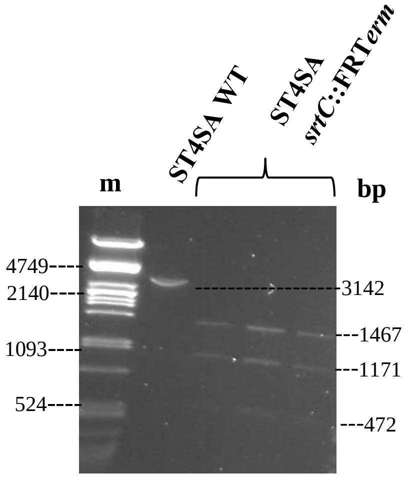

Additional file 1: Fig. S11. Gene deletion and integration via homologous recombination into the genome of *E. mundtii* ST4SA at the *srtC* locus to create *E. mundtii* ST4SA *srtC*::FRT*erm*. **(a)** Schematic representing the wild-type (WT) *E. mundtii* ST4SA *srtC* gene locus and the recombinant *srtC* deletion and FRT-*erm* integration site. Boxed regions show the upstream (~ 1 kb) and downstream (~ 0.4 kb) regions of homology on the WT chromosome and the recombinant *srtC*::FRT*erm* locus. Cells harboring the *srtC* KO vector were selected on Cm and Em, followed by nisin induction for MazF toxin expression to select for mutants that have lost the plasmid backbone bearing *cat* and *mazF* genes. Double crossover mutants were selected and screened by PCR using the indicated primer combinations. **(b)** PCR amplification of WT (3142 bp) and *srtC* deletion and insertion mutants (3183 bp) using the primer pairs indicated in panel B, followed by restriction digestion of the amplicons with XbaI. The WT amplicon contained one *Xba*I restriction site, while the *srtC* mutant amplicons contained three *Xba*I restriction sites. **(m)** Lambda DNA digested with *Pst*I (NEB). Digested amplicons from one WT and three *srtC*::FRT*erm* insertion mutant colonies are shown.

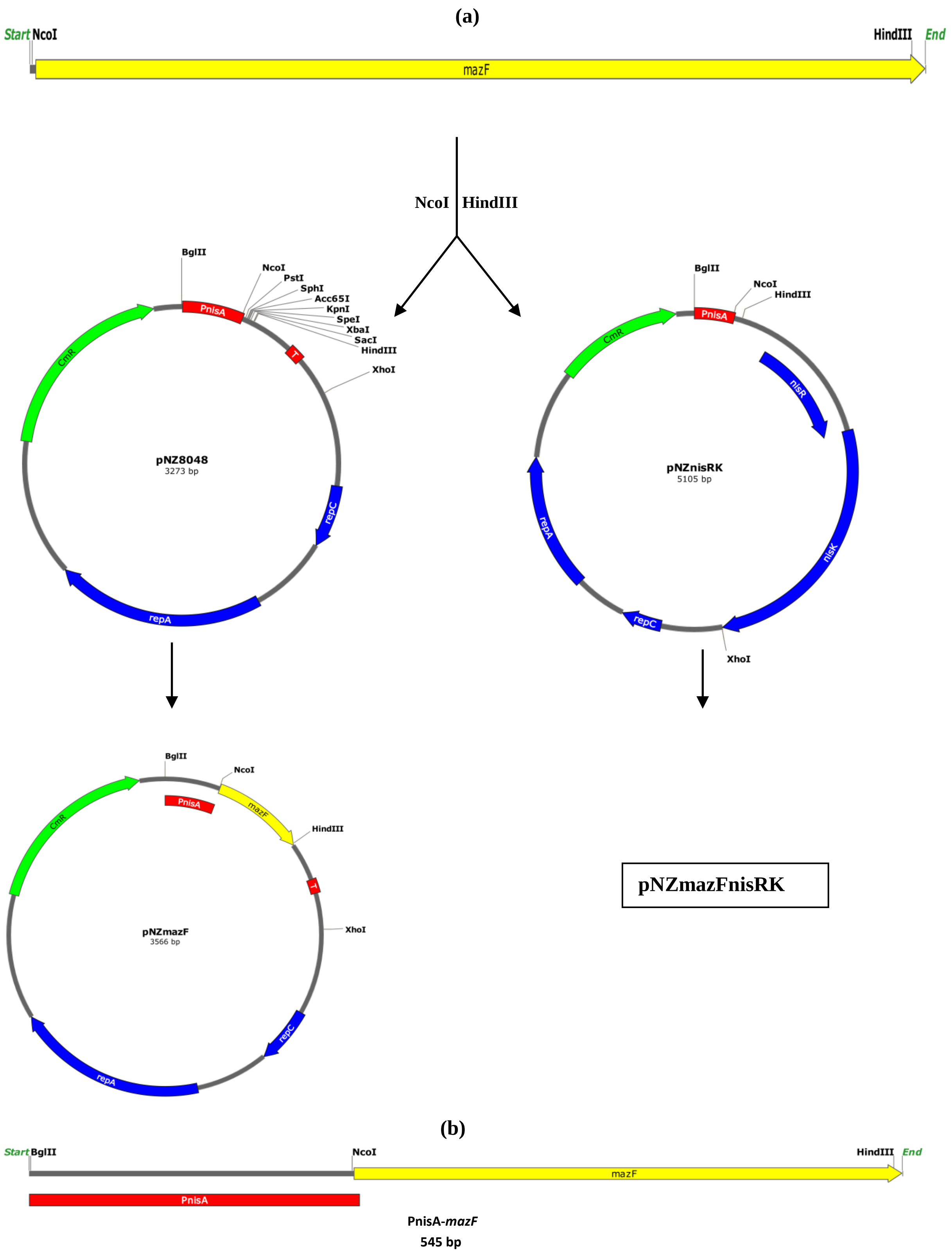

Additional file 1: Fig. S12. (a) Schematic representing the construction of the MazF toxin expression plasmids pNZmazF, pNZmazFnisRK and (b) the arrangement of PnisA promoter placed upstream of the *mazF* gene. Relevant restriction sites are shown. Construction of the plasmid is best understood by referring to “Materials and Methods” in the main text.

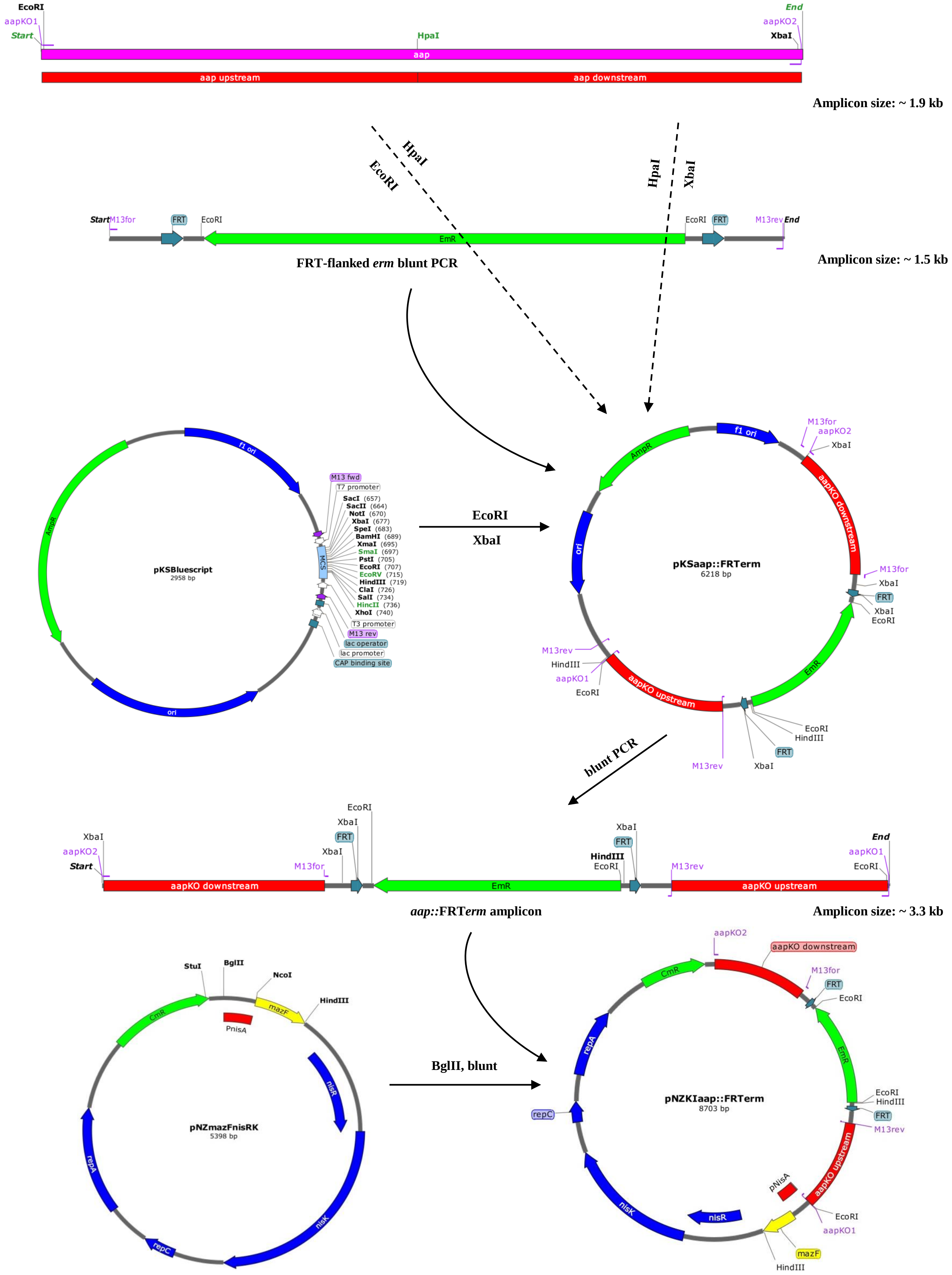
EcoRI
aapKO1
Start
HpaI
aap
End
aapKO2
XbaI
aap upstream
aap downstream
Amplicon size: ~ 1.9 kb
HpaI
EcoRI
HpaI
XbaI
Start M13for
FRT
EcoRI
EmR
EcoRI
FRT
M13rev End
FRT-flanked erm blunt PCR
Amplicon size: ~ 1.5 kb
pKSBluescript
2958 bp
f1 ori
AmpR
ori
M13 fwd
T7 promoter
SacI (657)
SacII (664)
NotI (670)
XbaI (677)
SpeI (683)
BamHI (689)
XmaI (695)
SmaI (697)
PstI (705)
EcoRI (707)
EcoRV (715)
HindIII (719)
ClaI (726)
SalI (734)
HincII (736)
XhoI (740)
T3 promoter
M13 rev
lac operator
lac promoter
CAP binding site
MCS
EcoRI
XbaI
pKSaap::FRTerm
6218 bp
f1 ori
AmpR
ori
M13for
aapKO2
XbaI
aapKO downstream
M13for
XbaI
FRT
XbaI
EcoRI
EmR
M13rev
HindIII
aapKO1
EcoRI
aapKO upstream
EcoRI
HindIII
FRT
M13rev
XbaI
blunt PCR
XbaI
aapKO2
Start
aapKO downstream
M13for
XbaI
FRT
XbaI
EcoRI
EmR
XbaI
FRT
HindIII
EcoRI
M13rev
aapKO upstream
End
aapKO1
EcoRI
aap::FRTerm amplicon
Amplicon size: ~ 3.3 kb
pNZmazFnisRK
5398 bp
StuI
BglII
NcoI
HindIII
CmR
mazF
PnisA
nisR
nisK
repC
repA
BglII, blunt
pNZKIaap::FRTerm
8703 bp
aapKO2
aapKO downstream
CmR
M13for
FRT
EcoRI
EmR
EcoRI
HindIII
FRT
M13rev
aapKO upstream
EcoRI
aapKO1
mazF
HindIII
pNisA
nisR
nisK
repC
repA

Additional file 1: Fig. S13. Schematic representing the construction of the pNZKIaap::FRTerm integrative plasmid containing the *erm* gene and two flanking sequences of the *aap* mucus adhesion gene from *L. plantarum* 423. Relevant genes, restriction sites and PCR primers are shown. Construction of the plasmid is best understood by referring to "Materials and Methods" in the main text.

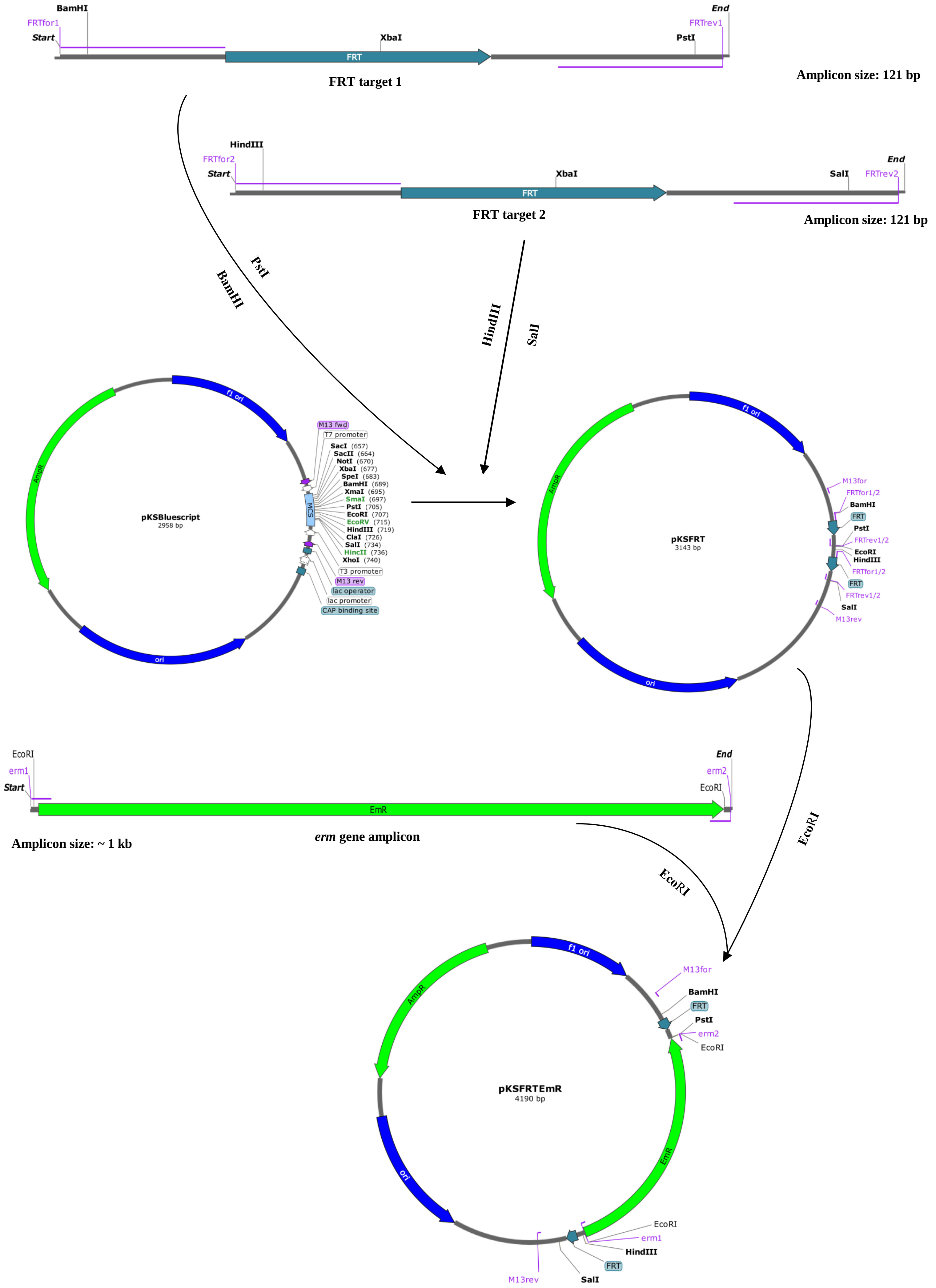
BamHI
FRTfor1
Start
XbaI
End
FRTrev1
PstI
FRT
FRT target 1
Amplicon size: 121 bp
HindIII
FRTfor2
Start
XbaI
End
SalI
FRTrev2
FRT
FRT target 2
Amplicon size: 121 bp
PstI
BamHI
HindIII
SalI
pKSBluescript
2958 bp
f1 ori
AmpR
ori
MCS
M13 fwd
T7 promoter
SacI (657)
SacII (664)
NotI (670)
XbaI (677)
SpeI (683)
BamHI (689)
XmaI (695)
SmaI (697)
PstI (705)
EcoRI (707)
EcoRV (715)
HindIII (719)
ClaI (726)
SalI (734)
HincII (736)
XhoI (740)
T3 promoter
M13 rev
lac operator
lac promoter
CAP binding site
pKSFRT
3143 bp
f1 ori
AmpR
ori
M13for
FRTfor1/2
BamHI
FRT
PstI
FRTrev1/2
EcoRI
HindIII
FRTfor1/2
FRT
FRTrev1/2
SalI
M13rev
EcoRI
erm1
Start
EmR
End
erm2
EcoRI
Amplicon size: ~ 1 kb
erm gene amplicon
EcoRI
EcoRI
pKSFRTEmR
4190 bp
f1 ori
AmpR
ori
EmR
M13for
BamHI
FRT
PstI
erm2
EcoRI
EcoRI
erm1
HindIII
FRT
M13rev
SalI

Additional file 1: Fig. S14. Schematic representing the construction of plasmid pKSFRTErm containing the FRT-flanked *erm* gene. Relevant genes, restriction sites and PCR primers are shown. Construction of the plasmid is best understood by referring to “Materials and Methods” in the main text.

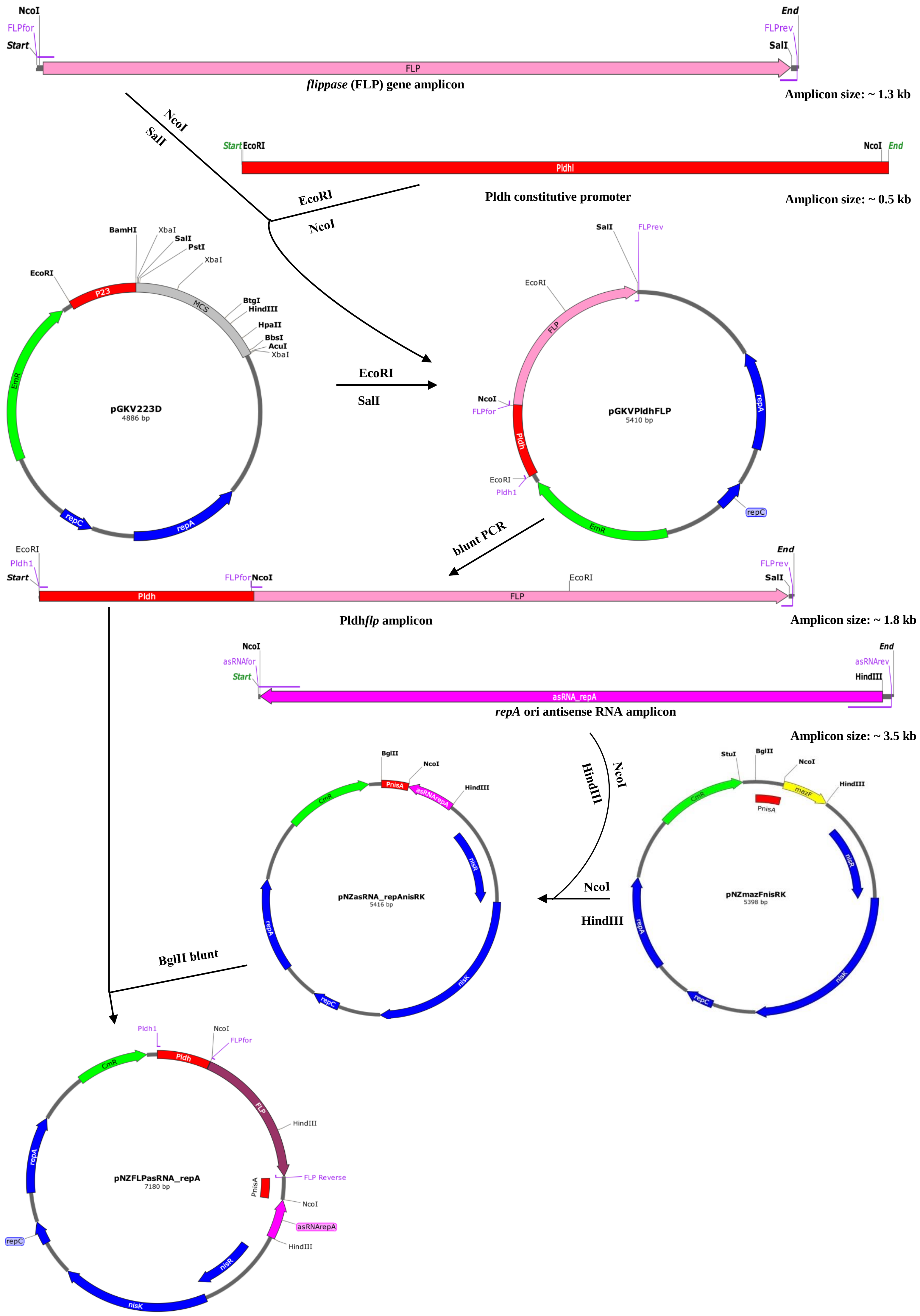
NcoI
FLPfor
Start
End
FLPrev
SalI
FLP
flippase (FLP) gene amplicon
Amplicon size: ~ 1.3 kb
NcoI
SalI
Start
EcoRI
NcoI
End
Pldhl
EcoRI
NcoI
Pldh constitutive promoter
Amplicon size: ~ 0.5 kb
BamHI
XbaI
SalI
PstI
XbaI
EcoRI
P23
MCS
BtgI
HindIII
HpaII
BbsI
AcuI
XbaI
EmR
pGKV223D
4886 bp
repC
repA
EcoRI
SalI
SalI
FLPrev
EcoRI
FLP
NcoI
FLPfor
pGKVPldhFLP
5410 bp
repA
Pldh
EcoRI
Pldh1
repC
EmR
blunt PCR
EcoRI
Pldh1
Start
FLPfor
NcoI
EcoRI
End
FLPrev
SalI
Pldh
FLP
Pldhflp amplicon
Amplicon size: ~ 1.8 kb
NcoI
asRNAfor
Start
End
asRNArev
HindIII
asRNA_repA
repA ori antisense RNA amplicon
Amplicon size: ~ 3.5 kb
BglII
NcoI
PnisA
asRNArepA
HindIII
CmR
nisR
pNZasRNA_repAnisRK
5416 bp
repA
nisK
repC
NcoI
HindIII
NcoI
HindIII
StuI
BglII
NcoI
HindIII
CmR
mazF
PnisA
nisR
pNZmazFnisRK
5398 bp
repA
nisK
repC
BglII blunt
Pldh1
NcoI
FLPfor
CmR
Pldh
FLP
HindIII
repA
pNZFLPasRNA_repA
7180 bp
PnisA
FLP Reverse
NcoI
asRNArepA
HindIII
repC
nisR
nisK

Additional file 1: Fig. S15. Schematic representing the construction of plasmid pNZFLPasRNA_repA containing the *flp* recombinase gene and a *repA* asRNA fragment. Relevant genes, restriction sites and PCR primers are shown. Construction of the plasmid is best understood by referring to “Materials and Methods” in the main text.
